# EpiSmoker2: a robust epigenetic classifier for smoking status inference using Illumina EPIC methylation data

**DOI:** 10.1080/17501911.2026.2630841

**Published:** 2026-02-17

**Authors:** Tianyu Zhu, Teodóra Faragó, Sailalitha Bollepalli, Aino Heikkinen, Mikaela Hukkanen, Olli Raitakari, Terho Lehtimäki, Tellervo Korhonen, Jaakko Kaprio, Fang Fang, Kaitlyn G. Lawrence, Dale P. Sandler, Mari Roberts Spildrejorde, Kristina Gervin, Yanyu Pan, Ricardo Costeira, Jordana T Bell, Miina Ollikainen

**Affiliations:** a Minerva Foundation Institute for Medical Research, Helsinki, Finland; b Institute for Molecular Medicine Finland (FIMM), Helsinki Institute of Life Science (HiLIFE), University of Helsinki, Helsinki, Finland; c Institute of Biotechnology (BI), Helsinki Institute of Life Science (HiLIFE), University of Helsinki, Helsinki, Finland; d Centre for Population Health Research, University of Turku and Turku University Hospital, Turku, Finland; e Research Centre of Applied and Preventive Cardiovascular Medicine, University of Turku, Turku, Finland; f Department of Clinical Physiology and Nuclear Medicine, Turku University Hospital, Turku, Finland; g InFLAMES Research Flagship, University of Turku, Turku, Finland; h Department of Clinical Chemistry, Fimlab Laboratories, and Finnish Cardiovascular Research Center - Tampere, Faculty of Medicine and Health Technology, Tampere University, Tampere, Finland; i GenOmics and Translational Research Center, RTI International, Research Triangle Park, NC, USA; j DLH, LLC, Bethesda, MD, USA; k Epidemiology Branch, National Institute of Environmental Health Sciences, National Institutes of Health, Research Triangle Park, NC, USA; l Department of Research and Innovation, Division of Clinical Neuroscience, Oslo University Hospital, Oslo, Norway; m Center for Treatment of Rheumatic and Musculoskeletal Diseases (REMEDY), Diakonhjemmet Hospital, Oslo, Norway; n Department of Twin Research and Genetic Epidemiology, King’s College London, London, UK

**Keywords:** DNA methylation, smoking status, biomarkers, LASSO regression, classifier, illumina EPIC array

## Abstract

**Aim:**

Tobacco smoking induces persistent DNA methylation (DNAm) changes in blood that can serve as long-term biomarkers for smoking exposure. We aimed to develop and validate a DNAm classifier of smoking status using Illumina EPIC array data.

**Methods:**

We built Epigenetic Smoking status Estimator2 (EpiSmokEr2), a Least Absolute Shrinkage and Selection Operator (LASSO) regression-based DNAm classifier using 511 CpGs from Illumina Infinium MethylationEPIC array (EPIC) data. The model was trained on 1343 samples from the Young Finns Study cohort and validated across six independent datasets from four cohorts and two array platforms (EPIC and EPICv2).

**Results:**

EpiSmokEr2 achieved an average sensitivity of 0.87 and specificity of 0.86 in distinguishing current from never smokers. Predicted smoking status correlated strongly with established DNAm smoking scores and GrimAge, indicating its ability to capture biologically relevant smoking effects. Simulation analysis showed EpiSmokEr2 was robust for up to 10% missing CpGs.

**Conclusion:**

EpiSmokEr2 provides a reliable DNAm-based estimator of smoking status. It is available as an open-source R package on GitHub, facilitating broad use in epidemiological and clinical research.

## PLAIN LANGUAGE SUMMARY

Smoking leaves chemical marks on our DNA, like footprints that reveal a person’s smoking history. We developed EpiSmokEr2, a tool that reads these marks to tell whether someone currently smokes, used to smoke, or has never smoked. The tool was built using data from Finnish participants and tested in other populations, where it also worked well. EpiSmokEr2 is free to use, and researchers can use it to uncover smoking exposure when people cannot or do not report it accurately. In the future, this tool could also help doctors check a patient’s smoking status.

## 1. Introduction

Tobacco smoking is a leading risk factor for cardiovascular disease, respiratory disorders, and multiple cancers [1–3]. While self-reported smoking status is widely used in research and clinical practice, it is susceptible to recall bias (inaccurate memory of smoking history), and social desirability bias (deliberate misreporting of smoking status due to stigma) [4,5]. Although metabolic biomarkers such as blood or urine cotinine provide objective measures of recent tobacco exposure, their utility is limited to short-term detection within 24–72 hours since the last tobacco use due to their short half-life [6].

DNA methylation (DNAm) has emerged as a persistent, long-term biomarker of smoking exposure, capturing both current and past smoking behavior in a dose-dependent manner [7–9]. Notably, hypomethylation at cg05575921 mapped to the gene body of *AHRR* (Aryl hydrocarbon receptor repressor) and other smoking-associated CpG sites can discriminate smokers from nonsmokers with area under the curves (AUCs) >0.9 and reflect the recency of cessation [8,10,11]. However, these scores are typically continuous measures and translating them into categorical smoking status (current/former/never smokers) requires thresholding. Due to variability in population characteristics, technological platforms and data processing methods, there is no such threshold that is universally applicable across datasets.

To address these challenges, we previously developed Epigenetic Smoking status Estimator (EpiSmokEr), a machine learning classifier designed for Illumina 450K methylation array data [7]. With the transition from 450K arrays to the more advanced EPIC and EPICv2 arrays, which cover ~900,000 and ~930,000 CpG sites, respectively, compared to 450K’s ~ 480,000 CpG sites, there is a growing need for updated classification tools. In this study, we introduce EpiSmokEr2, a new DNAm-based classifier designed for EPIC and EPICv2 data. We rigorously evaluated its performance in seven independent datasets across four cohorts comprising individuals from different ancestries (European and African) and provide it as an open-source R package to facilitate smoking classification in epidemiological and clinical research.

## 2. Methods

### 2.1. Selection and categorization of training samples

The training set is from the Young Finns Study (YFS) cohort [12,13], which includes EPIC samples from 1445 individuals with available smoking information [14]. A detailed description of the YFS dataset is provided in supplementary material. The smoking status at the time of sampling is obtained from self-reported questionnaires, based on the following question: *What is your current smoking status?*

1. Smokes once a day or more often
2. Smokes once a week or more often, but not daily
3. Smokes less often than once a week
4. Attempts to quit smoking
5. Has quit smoking
6. Has never smoked

Participants were categorized into 3 groups:
Never smokers (answer 6),
Former smokers (answer 5),
Current smokers (answers 1, 2, and 4).

Individuals who reported smoking less than once a week (answer 3, occasional smokers) were excluded from the training sample to reduce ambiguity in smoking status, as their DNAm profile (proxied by cg05575921 mapped to *AHRR* gene body [15]) was much closer to those of never smokers than current smokers (Figure S1). Self-reported passive smokers, as well as never smokers who have reported smoking in previous questionnaires were also removed. The final training set consisted of 1343 samples (current-smoker: 274; former-smoker: 337; never-smoker: 732).

### 2.2. Training of EpiSmoker2

To reduce dimensionality and exclude uninformative probes, CpGs were first filtered based on variability, retaining 192,549 probes with variance above the 75th percentile (variance > 0.00153) within the training dataset. A multinomial LASSO regression model was then trained using *glmnet* R package [16,17], including sex as an unpenalized covariate. The penalization parameter (λ) was determined via cross-validation by minimizing the multinomial deviance, following the same procedure described for EpiSmokEr [7]. The final model selected 511 CpGs in a data-driven manner and estimated the weights based on the optimal λ value.

For classification, the model outputs the log-odds of a sample belonging to each of the 3 smoking statuses. These log-odds were converted to class probabilities using the soft-max function, ensuring that the probabilities sum to one across all classes for one individual. The final smoking status classification was assigned to the category with the highest posterior probability.

### 2.3. Validation of EpiSmoker2

We evaluated EpiSmokEr2 in seven independent datasets across four cohorts: The Finnish Twin Cohort (FTC) [18–20], The GuLF Study (GuLF) [21], TwinsUK [22,23], and the GeNeup Study [24]. A detailed description of these datasets is provided in the supplementary material. Using self-reported smoking status as the reference, we evaluated classification performance by calculating sensitivity and specificity for each smoking category, as well as overall accuracy. The balanced accuracy of each smoking category was calculated as the mean of sensitivity and specificity. The metrics were calculated using the *confusionMatrix* function from *caret* R package [25]. We assessed performance in both 3-class (current, former, never smokers) and 2-class (current vs. never smokers) frameworks, excluding former smokers in the latter due to their ambiguous classification and potential overlap with other groups.

### 2.4. DNA methylation GrimAge2 and smoking pack-years calculation

DNAm GrimAge2 [26] is an epigenetic biomarker of aging based on DNA methylation patterns. It was calculated based on 9 DNAm-based surrogates of plasma proteins, an estimator of smoking pack-years (DNAm PACKYRS) and 2 demographic characteristics: chronological age and sex. Age acceleration (AgeAccelGrim2) was determined as the residuals from regressing DNAm GrimAge2 on chronological age. We correlated the EpismokEr2-predicted smoking status with AgeAccelGrim2 as well as DNAm PACKYRS, following established protocols [26].

### 2.5. Evaluation of EpiSmokEr2 robustness to missing CpGs

To assess the robustness of EpiSmokEr2 to missing CpGs in the input data, we systematically introduced missing values by randomly excluding 8% to 50% of CpGs in the FT12&16 dataset, covering a range above the baseline missing rate of 7% observed in the original quality-controlled beta matrix. For each missingness percentage (8%, 9%, 10%, 15%, 20%, 30%, 50%), we performed 50 independent iterations of random CpG exclusion and subsequent classification. Model performance was evaluated by comparing the overall accuracy of both 3-class (current/former/never smoker) and 2-class (current/never smoker) classifications against the baseline performance (7% missing CpGs).

## 3. Results

### 3.1. EpiSmoker2 model

**Figure 1.**
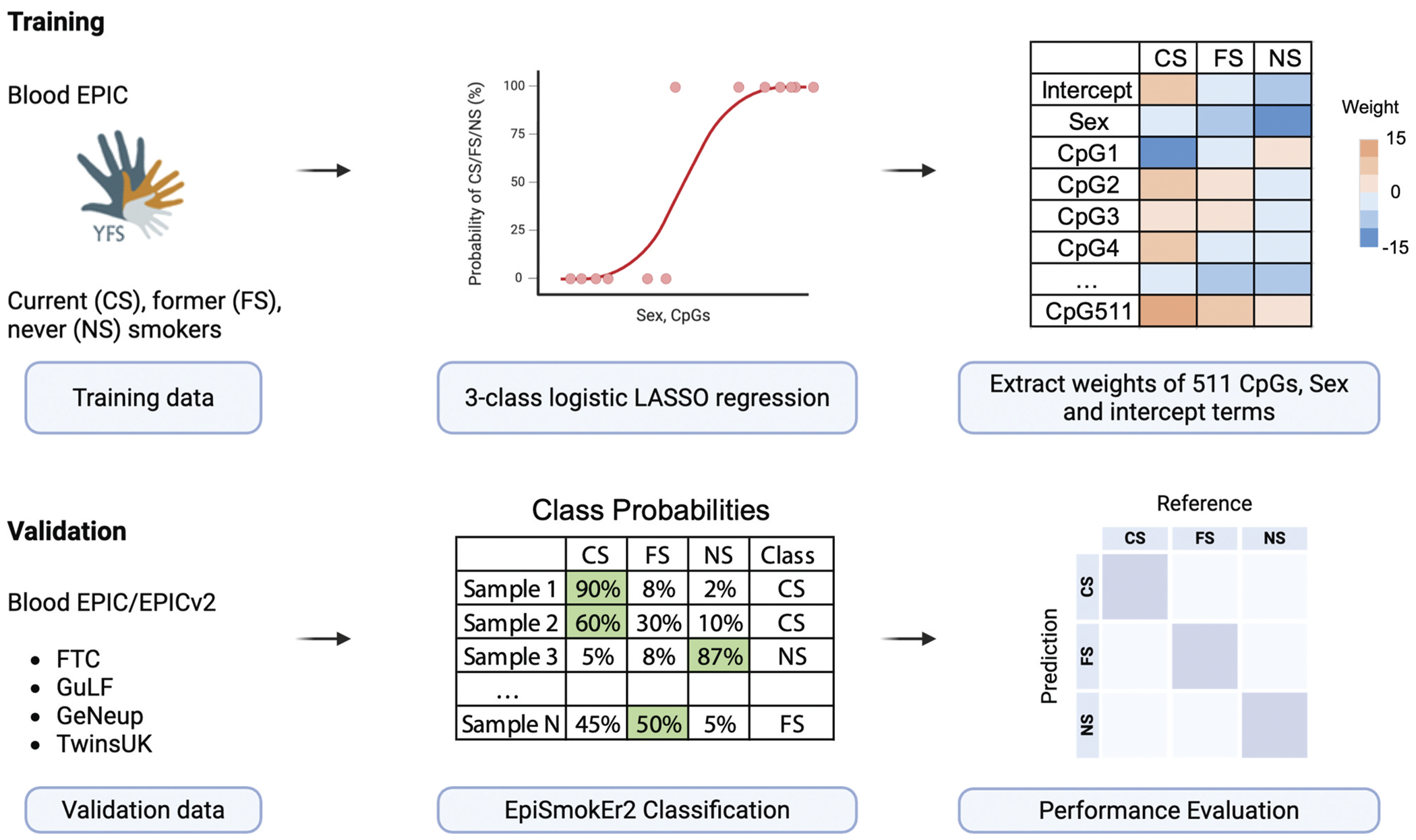
EpiSmokEr2 study overflow. EpiSmokEr2 was trained on 1343 EPIC samples from the Young Finns Study (YFS) Cohort, with smoking status (current, former, and never) determined by self-reported questionnaires. A 3-class logistic regression model with LASSO penalty (including sex as a fixed covariate) selected 511 discriminative CpGs. For validation, we applied the model to seven independent EPIC and EPICv2 data from four cohorts, assigning smoking status based on the highest predicted probability. Performance was assessed using sensitivity and specificity for each smoking category, as well as overall accuracy. Partially created in BioRender. Zhu, T. (2026) https://BioRender.com/yku3y2g.

An overview of workflow is shown in Figure 1. We trained a 3-class LASSO logistic regression model using blood sample DNAm EPIC data from the YFS, with self-reported smoking status. The final model is based on 511 CpGs, a sex and an intercept term (Figure S2). Top CpGs with the highest coefficients were mapped to *AHRR* gene body (cg05575921, cg26703534) and an intergenic smoking-related CpG (cg21566642). These CpGs were enriched in unannotated genomic regions and were underrepresented in TSS200 (two-sided Fisher’s exact test, FDR = 4e-6) and first exon regions (two-sided Fisher’s exact test, FDR = 8e-3, Figure S3).

### 3.2. Validation in EPIC datasets

**Figure 2.**
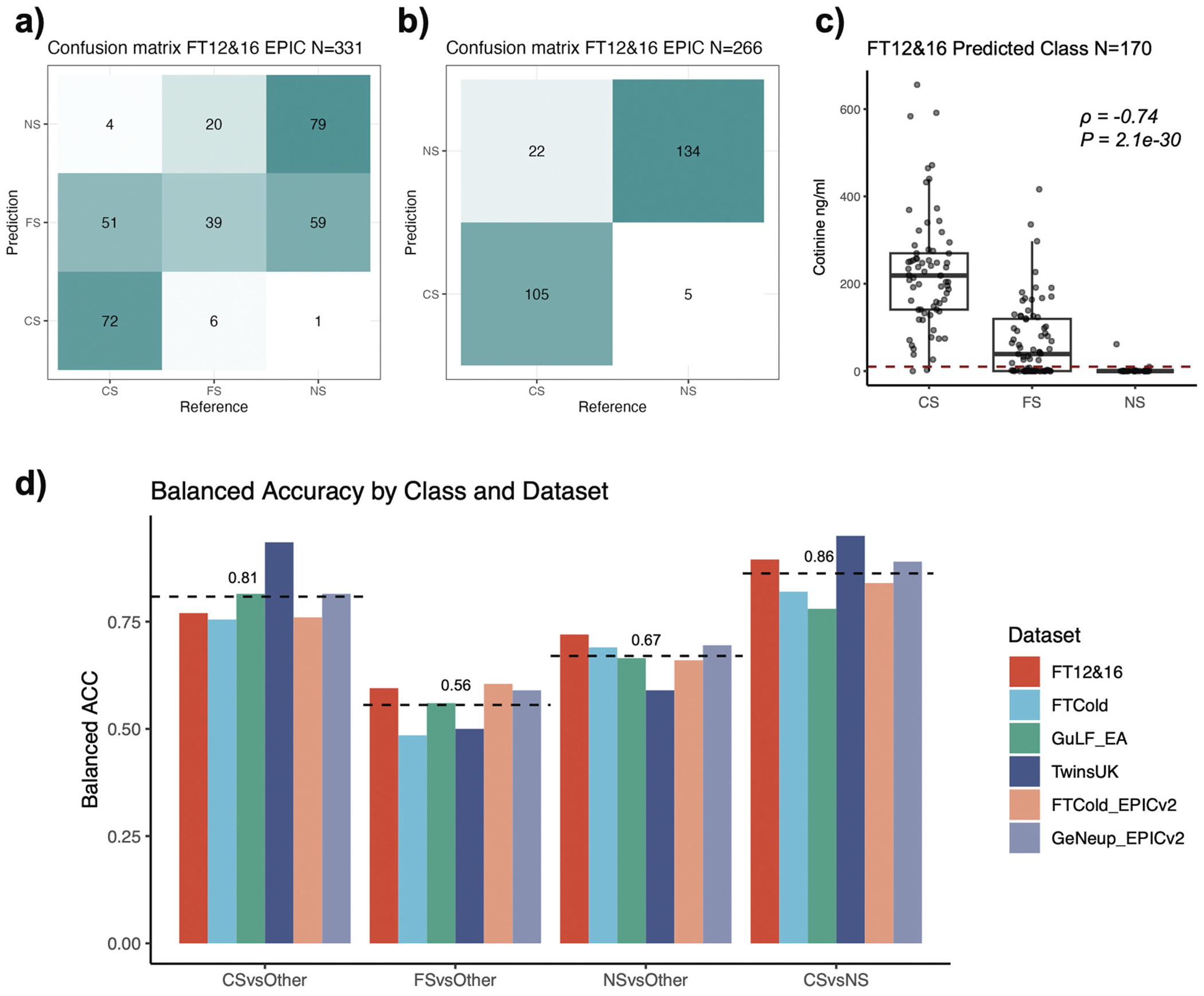
Validation of EpiSmokEr2. (a) Confusion matrix for EpiSmokEr2 applied to 331 FT12&16 EPIC samples. Numbers represent sample counts with reference (self-reported) smoking status (x-axis) versus predicted status (y-axis). (b) the same as (a), but excluding self-reported former smokers. Predictions were based on probability comparisons between current and never smokers. (c) Boxplot of cotinine levels in predicted current, former and never smokers. The red dashed line (10 ng/ml) indicates the threshold above which individuals are considered to have smoked within 24 hours before blood draw. Spearman’s rank correlation coefficients (ρ) and *p*-values were calculated between cotinine levels and the predicted smoking status, with current smokers (CS), former smokers (FS), and never smokers (NS) coded as 1, 2, and 3, respectively. (d) Barplot showing the balanced accuracy (defined as the mean of sensitivity and specificity) for predicting each class across six independent datasets (excluding the GuLF African ancestry dataset), using self-reported status as reference. CSvsOther, FSvsOther, and NSvsOther correspond to one-vs-rest evaluations in the 3-class classification. CSvsNS corresponds to 2-class classification of current versus never smokers. The black dashed line shows the mean balanced accuracy per smoking category. Colors indicate datasets. Abbreviations: CS, current smoker; FS, former smoker; NS, never smoker.

We evaluated our model across seven independent EPIC (v1 and v2) datasets from four cohorts: FTC, GuLF, TwinsUK, and GeNeup (Table 1, Methods). The confusion matrices showing the reference (self-reported) and predicted smoking status can be found in Figure 2(a,b), for FT12&16 FTC sub-cohort, and Figures S4–S9, for other cohorts. To assess the performance of our classifier, we calculated the sensitivity and specificity of classifying individuals into each category, as well as overall accuracy (Table 2, Methods). The 3-class classification only achieved average balanced accuracy (defined as average of sensitivity and specificity) of 0.81, 0.56 and 0.67 for current, former and never smokers respectively across all datasets, primarily due to misclassification of former smokers. When excluding the former smokers, the 2-class classification of current versus never smokers attained a higher average balanced accuracy of 0.86 (Figure 2(d)). Performance varied across populations, with one subset of the GuLF cohort (individuals of African ancestry) showed reduced accuracy compared to those of European ancestry (Table 2, Figure S6). Notably, the model performed well on datasets from the latest platform EPICv2 (Figure 2(d), Table 2).

Among samples with blood cotinine measurements in FT12&16 cohort (112 current, 34 former, and 24 never smokers), EpiSmokEr2-predicted smoking statuses were strongly correlated with cotinine levels (Spearman’s correlation coefficient ρ=−0.74, *p* = 2.1e-30). The predicted current smokers exhibited significantly higher cotinine levels compared to predicted never smokers, while predicted former smokers displayed intermediate levels, reflecting mixed profiles of current and never smokers (Figure 2(c)).

### 3.3. EpiSmoker2 predicts biological smoking status

To further explore the high misclassification rate among self-reported former smokers, we examined time since cessation, smoking duration, and pack-years in 60 samples from FT12&16 cohorts and 32 samples from FTC OLD cohort. Although these data were available only for a limited number of individuals, predicted smoking status showed consistent trends: self-reported former smokers with shorter cessation time (Spearman’s ρ=0.25, *p* = 0.054), marginally longer smoking duration (Spearman’s ρ=−0.21, *p* = 0.10), and higher pack-year (Spearman’s ρ=−0.50, *p* = 3.6e-3) were often classified as current smokers, whereas those with longer cessation time, shorter smoking duration, and lower pack-years were more often classified as never smokers (Figure S10).

**Figure 3.**
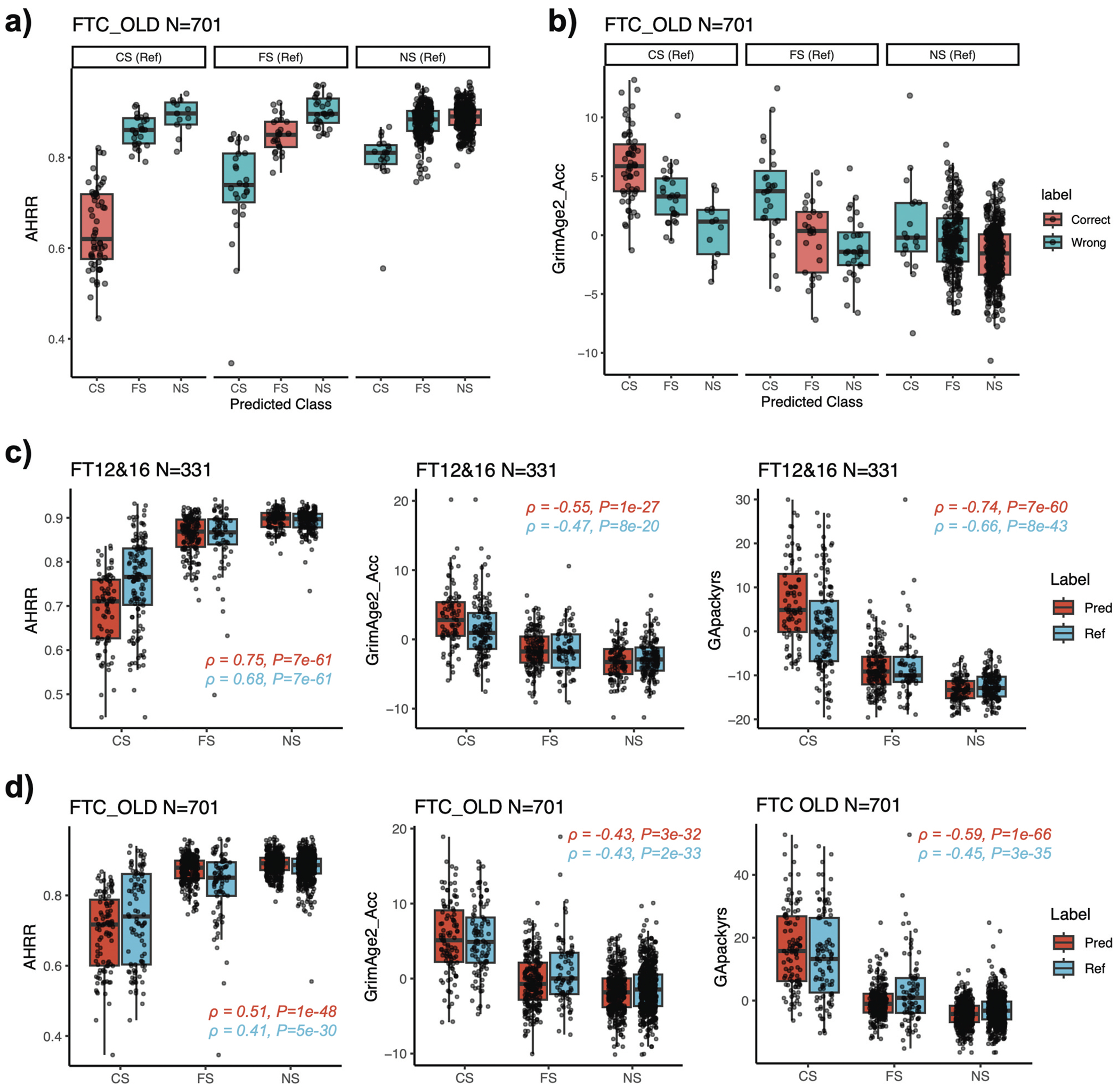
Correlation between EpiSmokEr2 predictions and DNAm biomarkers. (a) Boxplots showing *AHRR* (cg05575921) methylation levels in the FTC OLD cohort, stratified by reference (self-reported) smoking status (panels) and predicted smoking status (x-axis). Correctly classified samples are shown in red and misclassified samples in blue. (b) Same as (a), but for GrimAge2 age acceleration. (c) Boxplots showing the *AHRR* methylation (left), GrimAge2 age acceleration (middle) and GrimAge pack years (right) in the FT12&16 dataset. Predicted smoking status is shown in red and reference (self-reported) smoking status in blue. Spearman’s rank correlation coefficients (ρ) and *p*-values were calculated between the smoking-related scores and smoking status, with current smokers (CS), former smokers (FS), and never smokers (NS) coded as 1, 2, and 3, respectively. (d) Same as (c), but for FTC old cohort. Abbreviations: GrimAge2_Acc, GrimAge2 age acceleration; GApackyrs, GrimAge pack years; CS, current smoker; FS, former smoker; NS, never smoker.

We then correlated EpiSmokEr2 classifications with well-established biomarkers of smoking exposure: *AHRR* methylation [15] (smoking-related hypomethylation on cg05575921) and GrimAge2 [26] (an epigenetic clock related to smoking exposure). In the FTC OLD cohort, EpiSmokEr2-predicted smoking status consistently correlated with *AHRR* methylation, which was lowest in predicted current smokers, highest in predicted never smokers, and intermediate in former smokers, suggesting that methylation profiles in misclassified cases reflect biological smoking effects (Figure 3(a)). Similar trends were also observed across other independent cohorts (Figures S4–S9). Predicted current smokers also showed accelerated GrimAge2 aging compared to predicted former or never smokers (Figure 3(b)). Moreover, *AHRR* methylation, GrimAge2 age acceleration, and GrimAge2 pack-years showed stronger correlations with EpiSmokEr2 predictions than with self-reported status (Figure 3(c,d)), supporting its utility in capturing biologically meaningful smoking effects.

### 3.4. EpiSmoker2 is robust for up to 10% of missing CpGs

**Figure 4.**
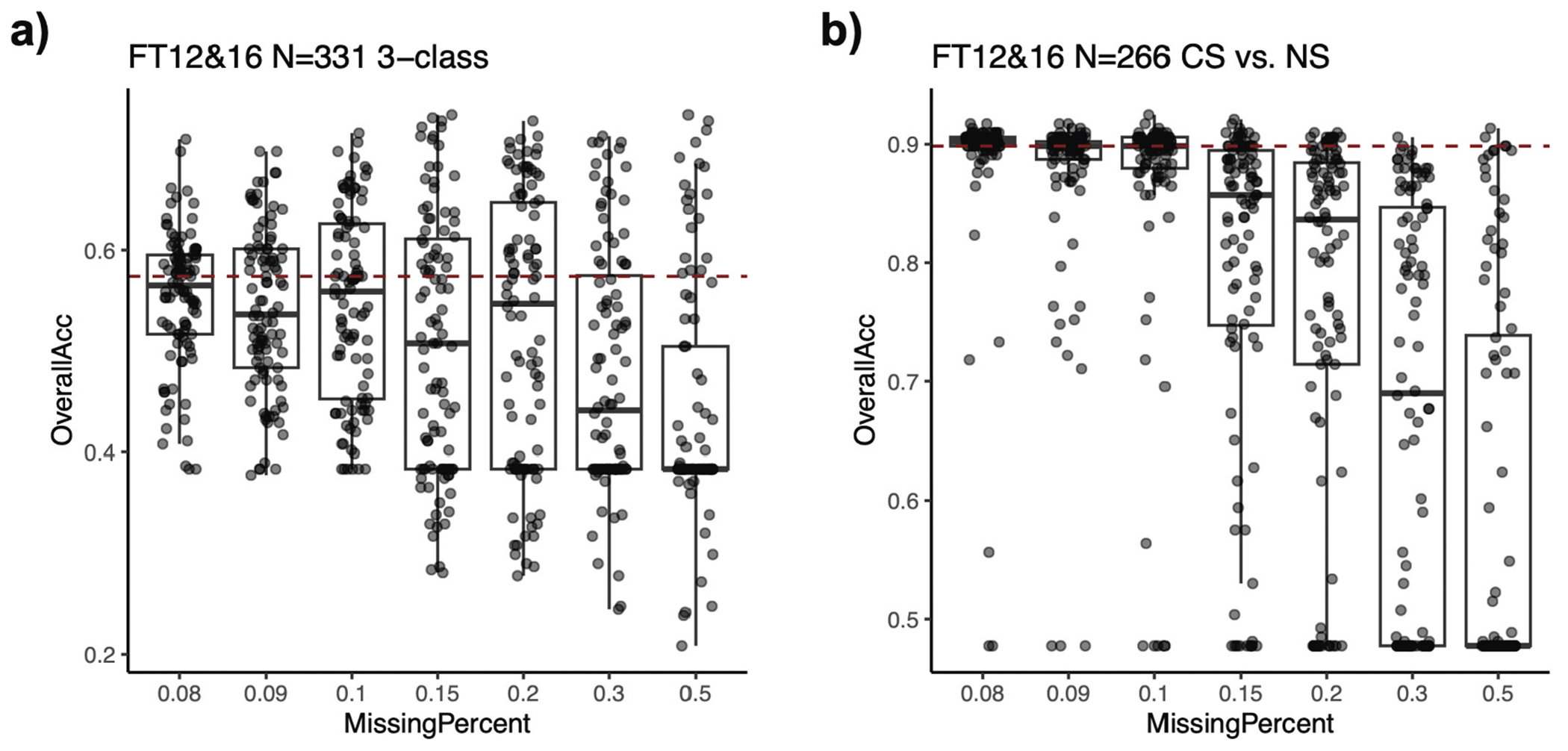
Robustness of EpiSmokEr2 under missing CpGs. (a) Boxplots showing the overall accuracy (y-axis) under varying percentages of missing CpGs (x-axis). For each percentage, CpGs were randomly masked for 50 times, and overall accuracy was derived from 3-class classification. The red dashed line indicates the baseline performance under 7% of missing CpGs. (b) Same as (a), but for 2-class classification of current and never smokers. Abbreviations: CS, current smoker; FS, former smoker; NS, never smoker.

A key advantage of EpiSmokEr2 is its incorporation of 511 CpG sites, which ensures robust performance even with partial missing data. To systematically evaluate this, we conducted simulations by artificially introducing varying proportions of missing CpGs (8%, 9%, 10%, 15%, 20%, 30%, 50%) in the FT12&16 test dataset. When compared to the baseline scenario (which contained ~7% missing CpGs), the model maintained high predictive accuracy with up to 10% missing CpGs, demonstrating its robustness to missing data (Figure 4(a,b)).

### 3.5. Comparison to EpiSmoker

The original EpiSmokEr was developed using Illumina 450K data and ultimately selected 121 CpGs through its model-fitting procedure [7]. Among them, only four CpGs were present in EpiSmokEr2 model, including highly weighted cg05575921 (mapped to *AHRR*) and cg21566642 (an intergenic smoking-related loci). Comparing the model performance of the two models on 450K and EPIC data respectively, we found that EpiSmokEr was better in 450K samples while EpiSmokEr2 remains optimal for EPIC samples (Figure S11). Together, the two tools provide complementary, platform-specific estimators aligned with the ongoing transition from 450K to EPIC in epigenetic epidemiology. The features of EpiSmokEr and EpiSmokEr2 are summarized in Table 3.

### 3.6. Performance comparison between male and female participants

To assess potential sex-specific differences in model performance, we applied EpiSmokEr2 separately to male and female participants in three FTC datasets (Table S1). In the FT12&16 datasets, where male and female sample sizes are relatively balanced, classification performance was highly comparable between sexes. In contrast, in the FTC EPICv1 and EPICv2 datasets, where there are more female participants, performance appeared higher in females than in males.

### 3.7. Classification of self-reported never smokers with inconsistent smoking histories

We also examined a subset of uncertain self-reported never smokers with inconsistent smoking histories – individuals who had previously reported smoking or who had documented passive exposure and were excluded from the primary analyses. EpiSmokEr2 classified 2 of 17 individuals in YFS and 18 of 32 individuals in FT12&16 as former smokers, and 1 FT12&16 individual as a current smoker (Figure S12).

## 4. Discussion

### 4.1. Performance and advantages of EpiSmoker2

We present EpiSmokEr2, an advanced DNAm-based classifier for smoking status that is compatible with EPIC and EPICv2 array data. Our model was rigorously validated in six independent European ancestry datasets across four cohorts, demonstrating robust performance in distinguishing current, former, and never smokers.

Performance in the FTC OLD cohort (EPIC and EPICv2) was slightly reduced compared with FT12&16 cohorts, likely due to differences in smoking status definition. In the FTC OLD cohort, missing smoking-status (*N* = 240) at the time of blood sampling was inferred from the questionnaire completed closest to the sampling date (within 10 years), introducing potential smoking status errors that may have impacted classification accuracy.

Performance was highly comparable between females and males in the FT12&16 dataset, where sample sizes were balanced. Although higher accuracy was observed in females than in males in the FTC OLD cohort (EPIC and EPICv2), this difference is more likely due to the substantially larger number of female participants and uneven distribution of smoking status across sexes, rather than a true sex-specific effect on model performance. Sex was explicitly included as a covariate to account for known sex-related differences in DNAm. While sex contributes to the model, the effect size is small relative to the major smoking-associated CpGs (Figure S2), indicating that the classification is primarily driven by smoking-related DNAm patterns rather than sex-specific effects.

EpiSmokEr2 offers two key advantages over existing methods. First, unlike conventional approaches that rely on DNAm scores which require arbitrary cutoffs to determine smoking status, EpiSmokEr2 directly outputs smoking status probabilities without the need for post-hoc thresholding. Supplementary analyses indicate broadly comparable performance between score-based classification using optimized thresholds and EpiSmokEr2 (Figure S13; Supplementary Methods). However, these approaches are not directly comparable, as DNAm scores are primarily intended to model smoking exposure as a continuous variable in association analyses, whereas EpiSmokEr2 is designed for robust categorical inference. We further note that although EpiSmokEr2 also outputs smoking probability that is well correlated with cotinine level (Figure S14), these probabilities are best interpreted as complementary measures reflecting classification uncertainty rather than precise estimates of smoking exposure. Second, like the first version, EpiSmokEr2 leverages a machine learning framework incorporating a large number of CpG sites compared to previous score-based methods. This not only enhances predictive power but also ensures robustness in the presence of missing CpGs – a common issue in real-world datasets due to probe filtering or platform differences.

### 4.2. CpG features and biological relevance

Among the CpGs selected in EpiSmokEr2, the top-ranked sites overlapped with well-established smoking-associated loci identified in multiple epigenome-wide association studies (EWAS). For instance, cg05575921 and cg26703534 in the *AHRR* gene body and cg21566642 in an intergenic region have been repeatedly highlighted as robust smoking biomarkers [15,27–29]. While most of the remaining CpGs selected by the model were located in unannotated regions and showed no clear pathway enrichment, their selection suggests that they may capture biologically relevant signals not yet characterized. This highlights a key advantage of machine learning approaches in uncovering predictive features that appear random but may represent novel markers of interest.

### 4.3. Classification challenges in former smokers

A limitation of our model is the seemingly reduced accuracy in classifying self-reported former smokers. This limitation is likely due to varying definitions of smoking cessation (e.g., time since quitting, smoking intensity prior to cessation), social desirability to report cessation in a health-related survey despite continuing to smoke, possibility for passive smoking or active nicotine replacement therapy (NRT), and the dynamic nature of post-cessation DNAm changes. Thus, former smokers do not represent a biologically homogeneous group, creating substantial overlap between current and former smokers as well as between former and never smokers. Consistent with this interpretation, classification among former smokers was associated with time since cessation, smoking duration, and pack-years, indicating that discrepancies primarily reflect underlying exposure history rather than random model error. Importantly, the biological signal of smoking captured by EpiSmokEr2 may be more relevant for downstream health-risk assessment than strict agreement with self-reported smoking categories. This was further supported by the result that EpiSmokEr2-predicted smoking status showed stronger correlations with both *AHRR* methylation levels and accelerated epigenetic aging than self-reported smoking status. Harmonized and detailed smoking-history variables would further improve interpretability and classification of former smokers.

### 4.4. Ancestry considerations

While performance was excellent in European ancestry populations, predictive accuracy was reduced in individuals of African ancestry. The discrepancy likely stems from the European ancestry of our training data, as population-specific DNAm patterns have been well-documented for smoking-associated loci [30,31]. Although CpGs with known SNPs were removed from our training set, ancestry-related differences in methylation quantitative trait loci (meQTL) landscapes and baseline DNAm distributions may still influence the magnitude and direction of smoking-associated DNAm changes, thereby affecting model performance. However, many of the CpGs selected by the model map to well-established smoking-associated loci (e.g., *AHRR*) that have been replicated across diverse ancestries in previous epigenome-wide association studies [15,27–29,32]. This suggests that the core biological smoking signal is broadly conserved and that ancestry-specific DNAm effects are likely to be modest on smoking-related DNAm changes. In addition to ancestry-related biological differences, definitions of “current,” “former,” and “never” smoking status may vary across cultural or study-specific contexts [33]. Such variation can lead to label inconsistency between cohorts and likely contributed to the reduced performance. Achieving optimal cross-population performance may therefore require ancestry-specific calibration or harmonized phenotype definitions. Together, these findings emphasize the importance of incorporating more diverse populations into future training datasets.

### 4.5. Identification of misreporting in questionnaire

To further assess the utility of EpiSmokEr2 in situations where self-reported smoking history may be uncertain, we also evaluated a subset of uncertain self-reported never smokers from the YFS and FTC cohorts. EpiSmokEr2 classified a subset of them as former or current smokers, indicating the presence of detectable smoking-related DNAm signatures. While the true smoking status of these individuals cannot be definitively established due to potential inconsistencies in questionnaire data and the possibility of occasional (non-daily) or sporadic smoking, these results highlight that the classifier reflects underlying biological exposure rather than reliance on categorical self-report. This reinforces the value of DNAm-based classification, particularly in contexts where misreporting or recall bias may affect questionnaire-derived smoking status.

### 4.6. Future directions

Future predictions could involve further validation in larger, more diverse populations and exploration of longitudinal DNAm changes. Additionally, as single-cell and whole-genome sequencing technologies advance, adapting EpiSmokEr2 to these platforms may further expand its utility in both research and clinical settings.

## 5. Conclusion

EpiSmokEr2 provides a reliable DNAm-based approach for classifying smoking status across diverse cohorts and array platforms. It improves on traditional score-based methods by giving smoking status directly, using a wide set of CpG sites, and staying reliable even when some probes are missing. While former smokers remain challenging to classify because of heterogeneous cessation patterns, the tool nevertheless captures biologically meaningful smoking effects, as reflected in its strong association with time since cessation, duration of smoking, smoking pack-years, *AHRR* methylation and epigenetic aging. As an open-source and practical resource, EpiSmokEr2 offers an effective solution for enhancing smoking exposure assessment in epidemiological and clinical research.

## Supplementary Material

Supp 1

Supp 2

Supplemental data for this article can be accessed online at https://doi.org/10.1080/17501911.2026.2630841.

## Figures and Tables

**Table 1.** Statistics of the datasets in this study.

Dataset	Total	CS	FS	NS	Sex (F/M)	Age (mean±SD)	Race	Platform
YFS	1343	274	337	732	751/592	41.8 ± 5.1	European	EPIC
FT12&16	331	127	65	139	164/167	25.3 ± 2.2	European	EPIC
FTCOLDv1	701	95	80	526	641/60	65.6 ± 9.4	European	EPIC
GuLF_EA	843	302	181	360	0/843	46.0 ± 11.8	European	EPIC
GuLF_AA	632	214	75	343	0/632	41.4 ± 11.0	African	EPIC
TwinsUK	641	29	235	377	632/9	62.3 ± 9.6	European	EPIC
FTCOLDv2	223	52	69	102	145/78	57.5 ± 7.2	European	EPICv2
GeNeup	1054	185	520	349	498/556	57.5 ± 9.5	European	EPICv2

Abbreviations: SD = standard deviation; CS = current smoker; FS = former smoker; NS = never smoker.

**Table 2.** Validation Performance Metrics.

Dataset	Sensitivity	Specificity	Overall accuracy
FT12&16 (*N* = 331)			0.57
CS vs others	0.57	0.97	
FS vs others	0.60	0.59	
NS vs others	0.57	0.87	
CS vs NS	0.83	0.96	0.90
FTC OLD EPIC (*N* = 701)			0.57
CS vs others	0.59	0.92	
FS vs others	0.31	0.66	
NS vs others	0.61	0.77	
CS vs NS	0.73	0.91	0.88
GuLF_EA (*N* = 843)			0.58
CS vs others	0.99	0.64	
FS vs others	0.33	0.79	
NS vs others	0.37	0.96	
CS vs NS	0.99	0.57	0.76
GuLF_AA (*N* = 632)			0.37
CS vs others	0.96	0.44	
FS vs others	0.20	0.71	
NS vs others	0.04	0.99	
CS vs NS	1.00	0.17	0.49
TwinsUK_EPIC (*N* = 641)			0.63
CS vs others	0.90	0.97	
FS vs others	0	1	
NS vs others	1	0.18	
CS vs NS	0.90	1	0.99
FTC OLD EPICv2 (*N* = 223)			0.56
CS vs others	0.65	0.87	
FS vs others	0.58	0.63	
NS vs others	0.49	0.83	
CS vs NS	0.79	0.89	0.86
GeNeup_EPICv2 (*N* = 1054)			0.57
CS vs others	0.86	0.77	
FS vs others	0.50	0.68	
NS vs others	0.51	0.88	
CS vs NS	0.96	0.82	0.87

Predicted smoking status was compared with self-reported smoking status. For each smoking status category, the sensitivity and specificity values were calculated by comparing that one with the other two categories (3-class classification), and current to never smokers (2-class classification). Overall accuracy is the proportion of all correctly classified samples out of the total number of samples.

Abbreviations: CS = current smoker; FS = former smoker; NS = never smoker.

**Table 3.** Comparison of EpiSmokEr and EpiSmokEr2.

Category	EpiSmokEr	EpiSmokEr2
Array platform used in model training	Illumina 450K	Illumina EPIC
Training method	3-class LASSO regression	3-class LASSO regression
Number of CpGs selected	121 (94 available on EPIC)	511 (230 available on 450K)
Intended use	Optimal for 450K datasets	Optimal for EPIC/EPICv2 datasets
Limitations	Reduced performance on EPIC due to missing probes	Reduced performance on 450K due to missing probes

## Data Availability

YFS: The dataset supporting the conclusions of this article were obtained from the Cardiovascular Risk In Young Finns study which comprises health related participant data. The use of data is restricted under the regulations on professional secrecy (Act on the Openness of Government Activities, 612/1999) and on sensitive personal data (Personal Data Act, 523/1999, implementing the EU data protection directive 95/46/EC). Due to these restrictions, the data can not be stored in public repositories or otherwise made publicly available. Data access may be permitted on a case by case basis upon request only. Data sharing outside the group is done in collaboration with YFS group and requires a data-sharing agreement. Investigators can submit an expression of interest to the chairman of the publication committee (Prof Mika Kähönen, Tampere University, Finland). FTC: The phenotype and methylation data from the Finnish Twin Cohort used in this study are stored in the Biobank of the Finnish Institute for Health and Welfare (Helsinki, Finland). Qualified researchers may obtain these data through a standardized application procedure (https://thl.fi/en/statistics-and-data/data-and-services/research-use-and-data-permits). GuLF: The GuLF study welcomes collaborative research proposals. For details on access and proposal preparation, please visit: https://gulfstudy.nih.gov/en/forresearchers.html. Once approved, de-identified datasets needed to address specific research questions will be shared under an official NIH Data Transfer Agreement. These agreements ensure the confidentiality and privacy of study participants and usually require approval from both NIH and the recipient organization, as well as oversight from an Institutional Review Board. TwinsUK: The majority of TwinsUK DNA methylation data used in this study are uploaded on the ReShare UK Data Service, under Data collection identifier 853,526. Access to further individual-level data including DNA methylation and phenotype data can be applied for through the TwinsUK cohort data access committee. For information on access and how to apply, see https://twinsuk.ac.uk/researchers/access-data-and-samples/request-access/. GeNeup: The authors confirm that the data supporting the study’s findings are included within the article and its supplementary materials. Processed data may be shared upon reasonable request. Individual-level genetic data cannot be provided due to the ethical approvals governing this research.

## Article highlights

- Developed EpiSmokEr2, a DNA methylation-based classifier of smoking status compatible with Illumina EPIC and EPICv2 arrays.
- Validated in six independent European population datasets across four cohorts.
- Estimates probabilistic smoking status predictions, eliminating arbitrary cutoffs used in traditional score-based methods.
- Demonstrates robust performance, with high sensitivity/specificity and resilience to missing CpGs.
- Captures biologically relevant smoking effects, strongly correlating with time since cessation, duration of smoking, smoking pack-years, *AHRR* methylation and epigenetic aging measures.
- Available as a freely accessible open-source R package, supporting epidemiological and clinical research.
