# Supplementary material for: EpiSmoker2: a robust epigenetic classifier for smoking status inference using Illumina EPIC methylation data
Source: Epigenomics. Author manuscript; Available in PMC 2026 Mar 12. (PMC12962688; doi:10.1080/17501911.2026.2630841)
Supplement: Supp 1 [file NIHMS2149663-supplement-Supp_1.docx]

Supplementary figures


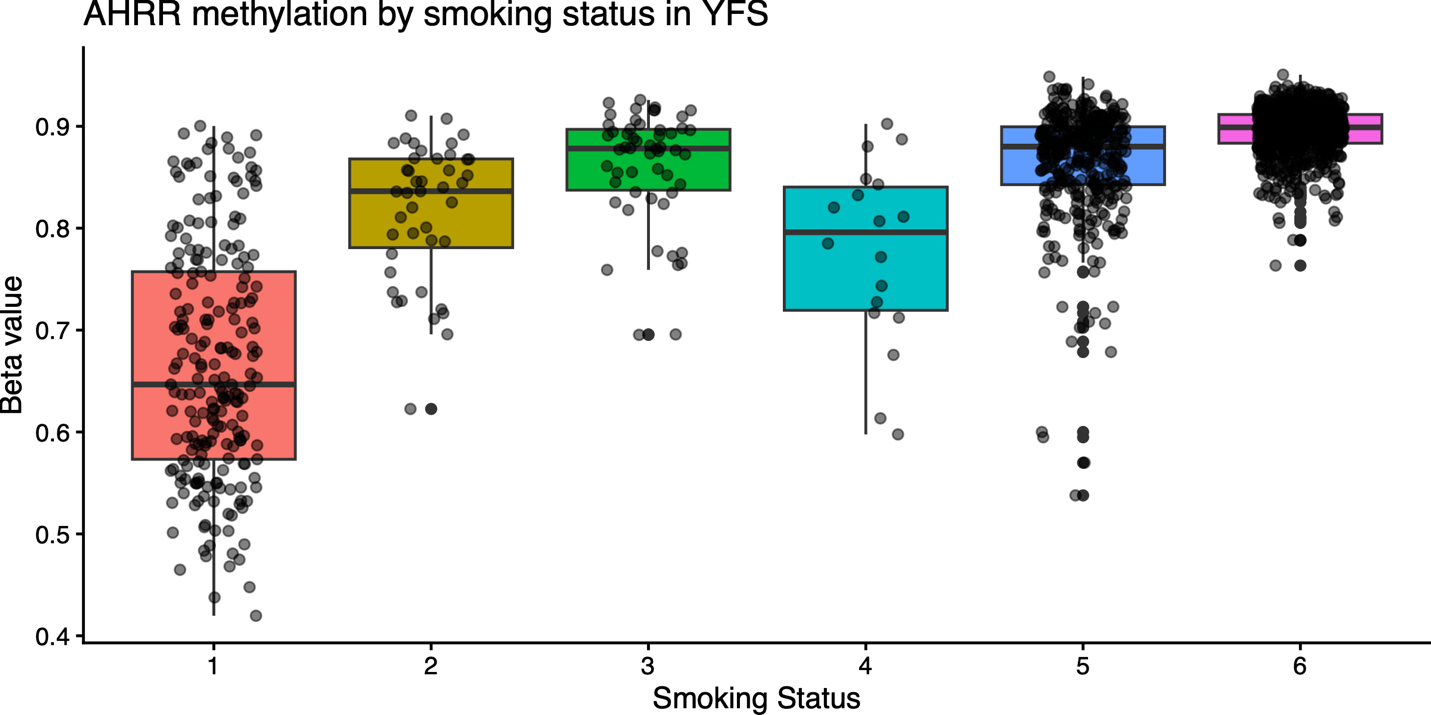


**Figure S1 AHRR methylation levels by smoking status in YFS data.** Boxplot showing the methylation beta values at cg05575921 (a well-established smoking-associated CpG site located in the gene body of AHRR) in YFS samples, stratified by self-reported smoking status. 1: Smokes once a day or more often; 2: Smokes once a week or more often, but not daily; 3: Smokes less often than once a week; 4: Attempts to quit smoking; 5: Has quit smoking; 6: Has never smoked.


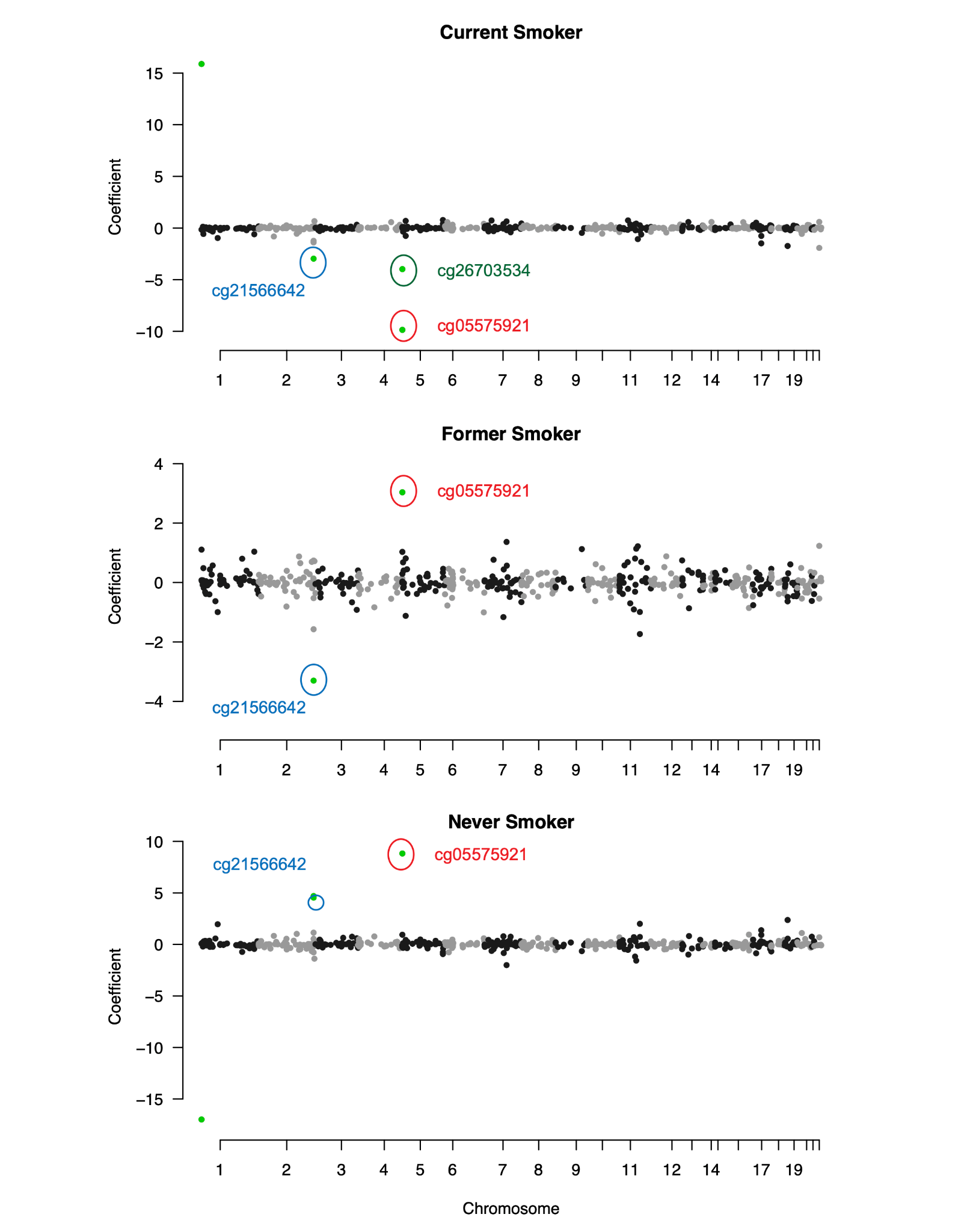


**Figure S2 Coefficients of all the features in EpiSmokEr2.** Scatterplot showing the regression coefficient for all 513 features in EpiSmokEr2, including the intercept (leftmost), sex term (second), and 511 CpG sites (ordered by chromosomal position). Established smoking-associated CpGs-cg05575921 (red), cg26703534 (green), and cg21566642 (blue) are highlighted.


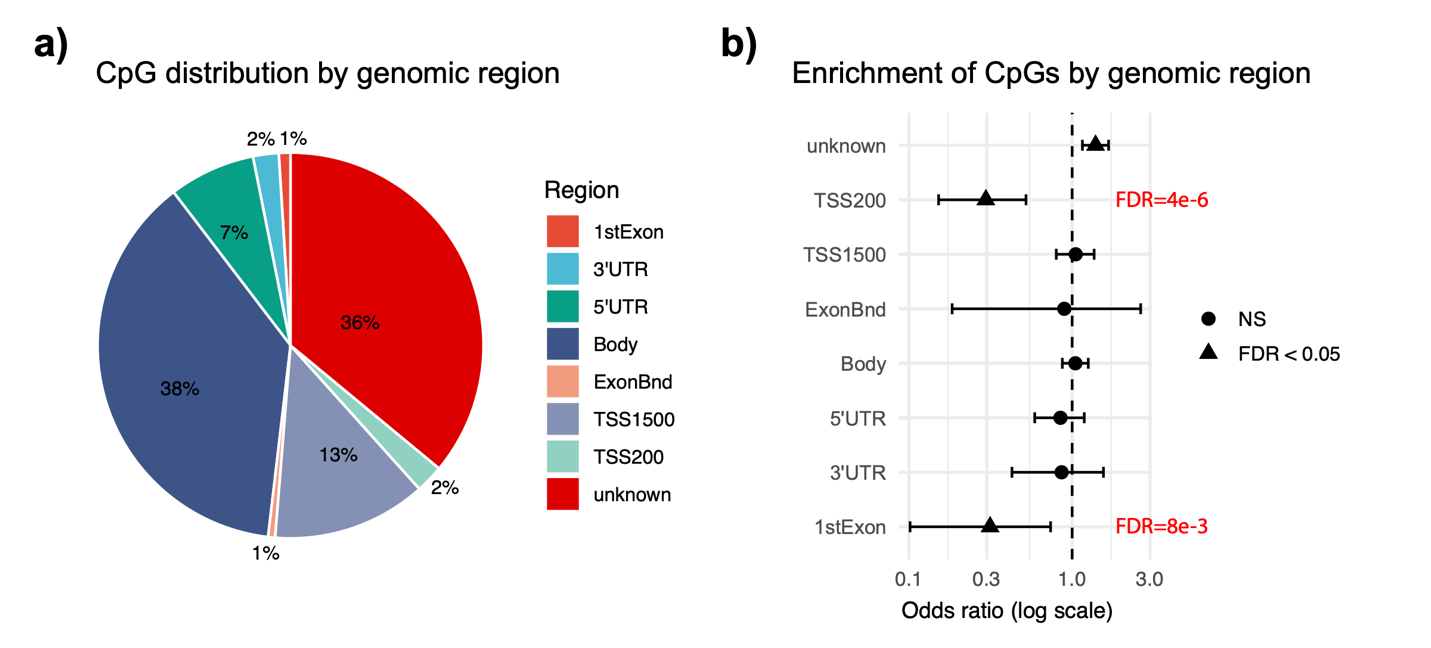


**Figure S3 Genomic distribution of EpiSmokEr2 CpGs. a)** Pie chart showing the percentage distribution of genomic regions to which the 511 CpGs in EpiSmokEr2 are mapped. **b)** Forest plot showing the enrichment of these genomic regions among the 511 EpiSmokEr2 CpGs, compared with all CpGs on the Illumina EPIC array as the background. Odds ratios (x-axis) were calculated using two-sided Fisher’s exact tests, with 95% confidence intervals indicated by horizontal bars. P-values were adjusted for multiple testing using the Benjamini-Hochberg method.


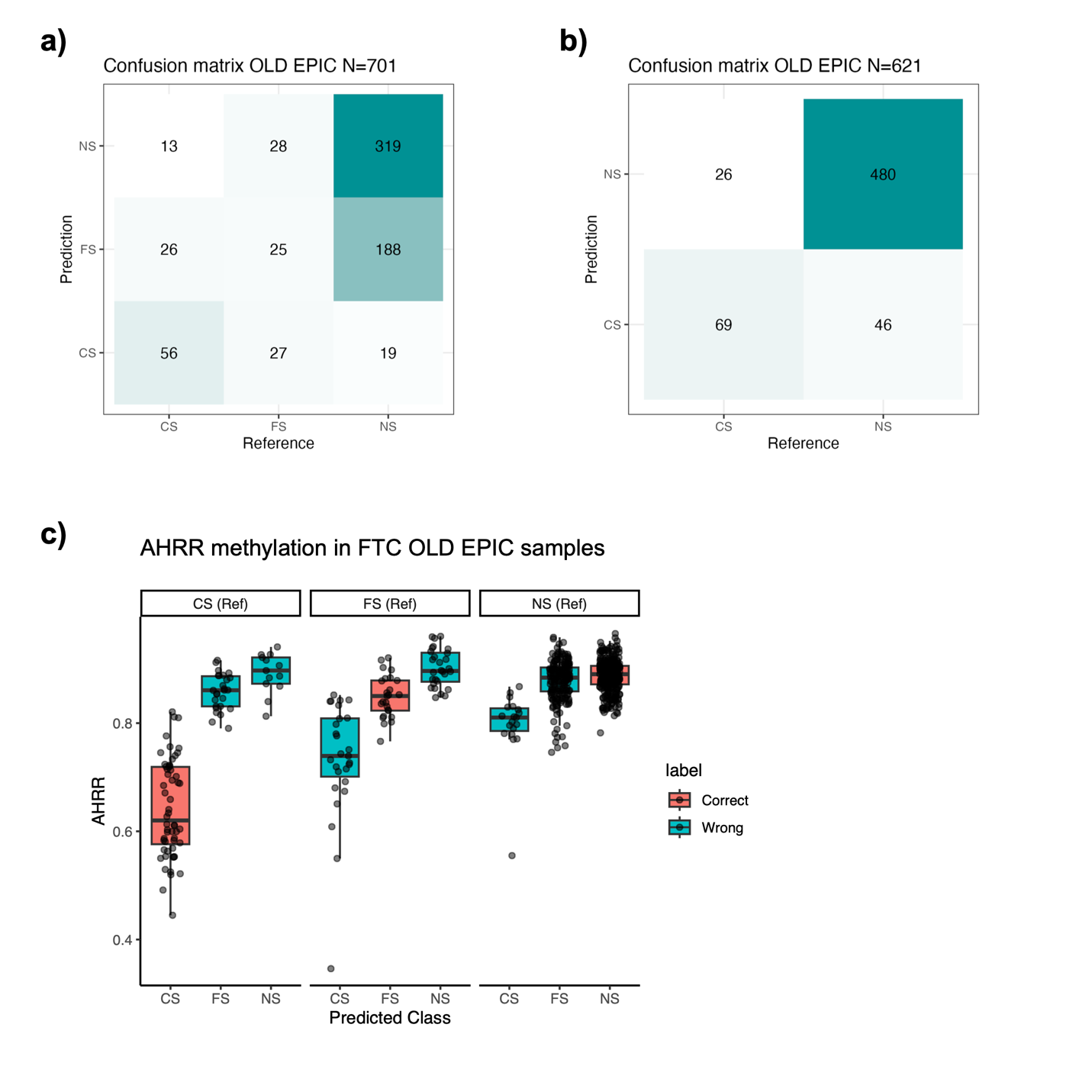


**Figure S4 Validation in FTC OLD EPIC samples.** **a)** Confusion matrix for EpiSmokEr2 applied to 701 FTC OLD EPIC samples. Numbers represent sample counts with reference (self-reported) smoking status (x-axis) versus predicted status (y-axis). **b)** the same as a), but excluding self-reported former smokers. Predictions were based on probability comparisons between current and never smokers. **c)** Boxplots showing AHRR (cg05575921) methylation levels in the FTC OLD EPIC samples. Stratified by reference (self-reported) smoking status (panels) and predicted smoking status (x-axis). Correctly predicted cases are shown in red; misclassified cases in blue.


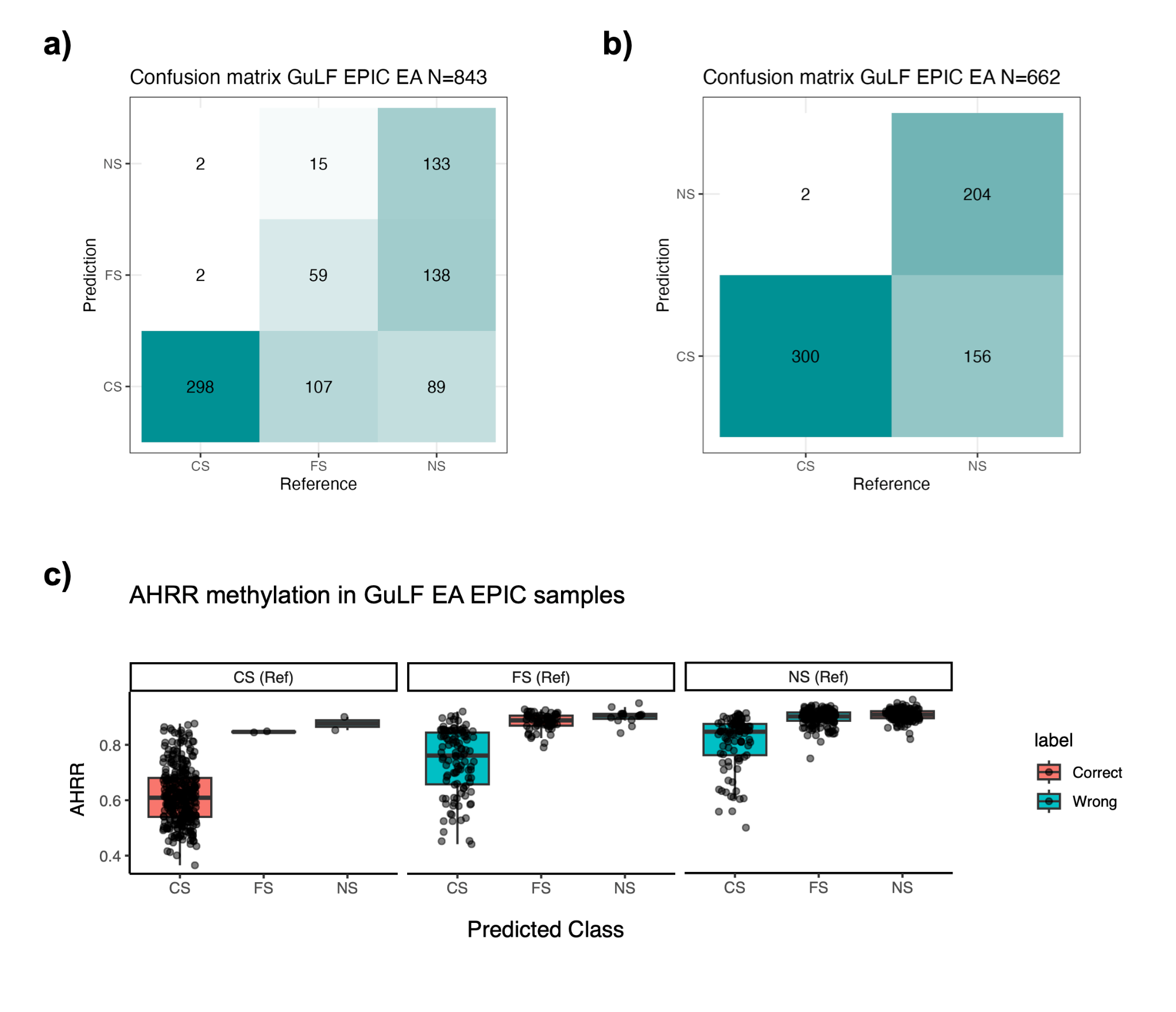


**Figure S5 Validation in GuLF European ancestry EPIC samples.** **a)** Confusion matrix for EpiSmokEr2 applied to 843 GuLF European ancestry EPIC samples. Numbers represent sample counts with reference (self-reported) smoking status (x-axis) versus predicted status (y-axis). **b)** the same as a), but excluding self-reported former smokers. Predictions were based on probability comparisons between current and never smokers. **c)** Boxplots showing AHRR (cg05575921) methylation levels in the GuLF European ancestry EPIC samples. Stratified by reference (self-reported) smoking status (panels) and predicted smoking status (x-axis). Correctly predicted cases are shown in red; misclassified cases in blue.


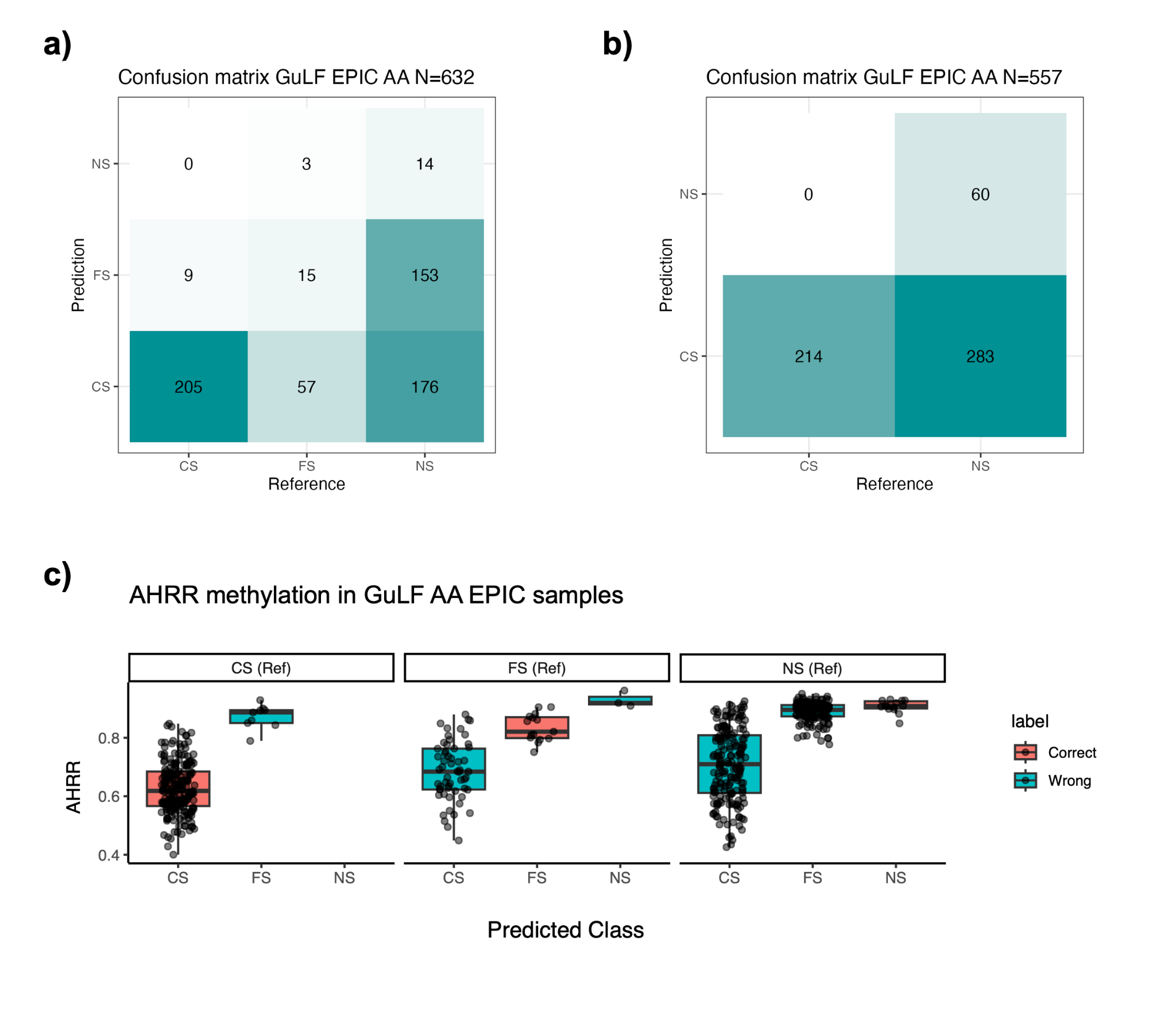


**Figure S6 Validation in GuLF African ancestry EPIC samples.** **a)** Confusion matrix for EpiSmokEr2 applied to 632 GuLF African ancestry EPIC samples. Numbers represent sample counts with reference (self-reported) smoking status (x-axis) versus predicted status (y-axis). **b)** the same as a), but excluding self-reported former smokers. Predictions were based on probability comparisons between current and never smokers. **c)** Boxplots showing AHRR (cg05575921) methylation levels in the GuLF African ancestry EPIC samples. Stratified by reference (self-reported) smoking status (panels) and predicted smoking status (x-axis). Correctly predicted cases are shown in red; misclassified cases in blue.


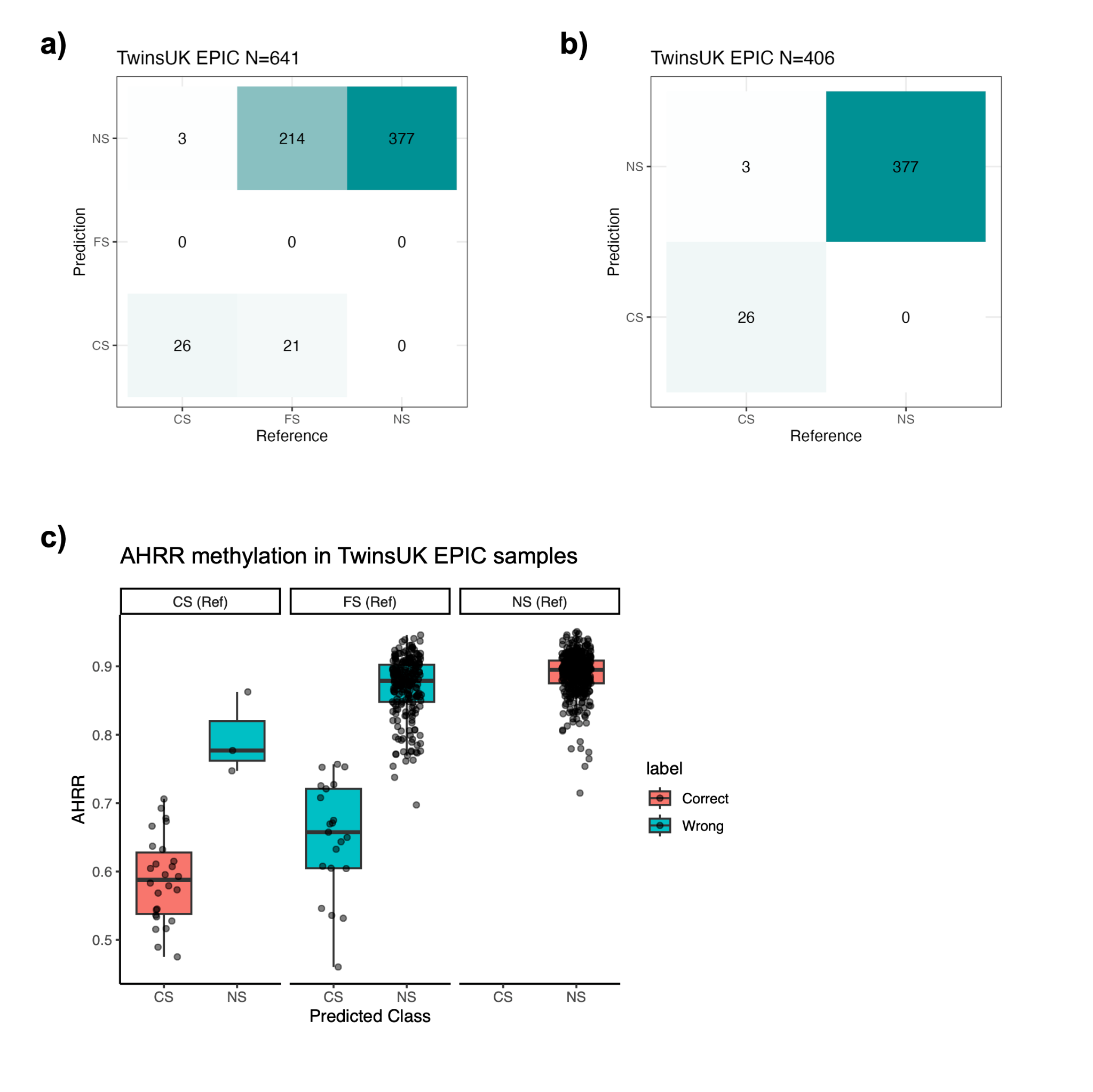


**Figure S7 Validation in TwinsUK EPIC samples.** **a)** Confusion matrix for EpiSmokEr2 applied to 641 TwinsUK EPIC samples. Numbers represent sample counts with reference (self-reported) smoking status (x-axis) versus predicted status (y-axis). **b)** the same as a), but excluding self-reported former smokers. Predictions were based on probability comparisons between current and never smokers. **c)** Boxplots showing AHRR (cg05575921) methylation levels in the TwinsUK EPIC samples. Stratified by reference (self-reported) smoking status (panels) and predicted smoking status (x-axis). Correctly predicted cases are shown in red; misclassified cases in blue.


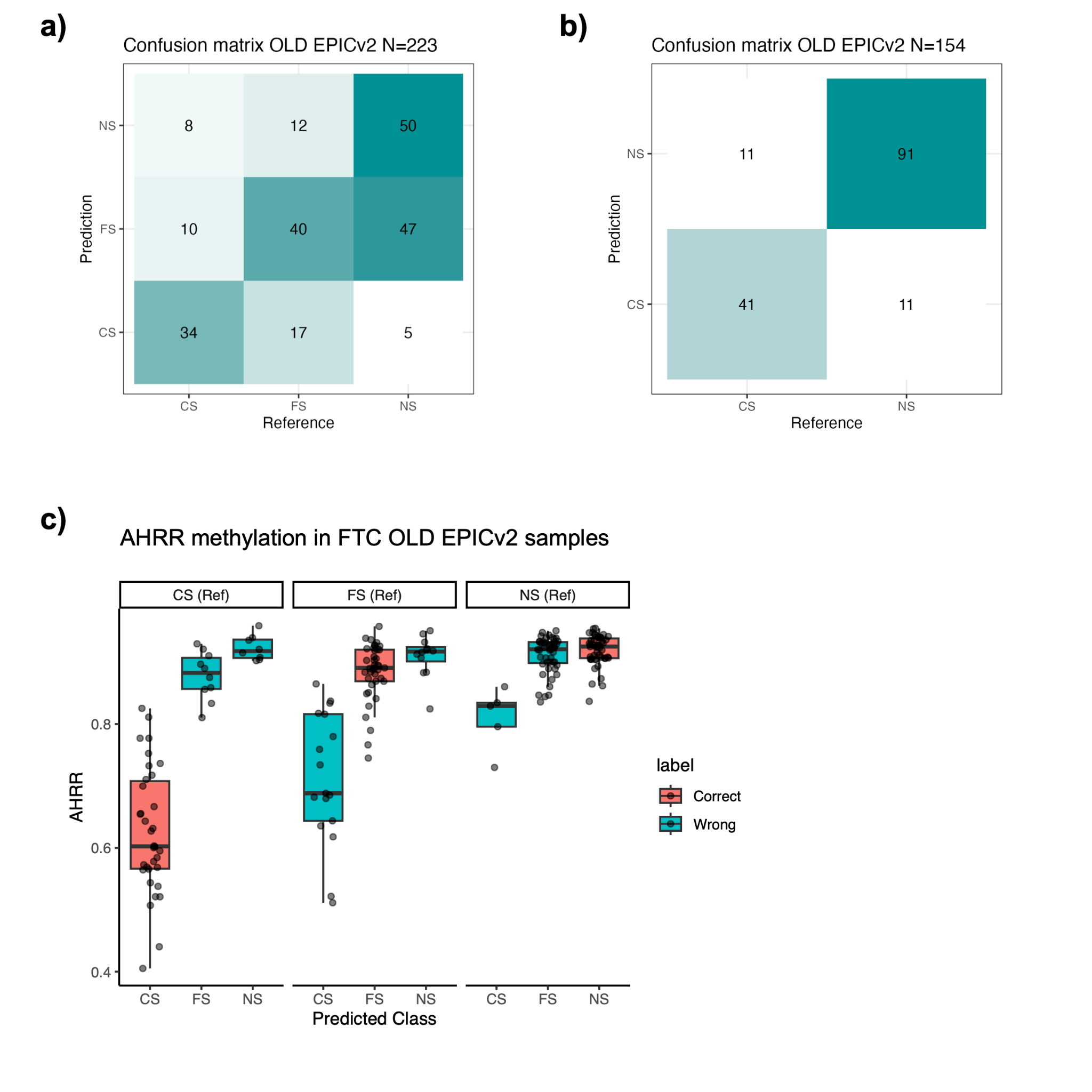


**Figure S8 Validation in FTC OLD EPICv2 samples.** **a)** Confusion matrix for EpiSmokEr2 applied to 223 FTC OLD EPICv2 samples. Numbers represent sample counts with reference (self-reported) smoking status (x-axis) versus predicted status (y-axis). **b)** the same as a), but excluding self-reported former smokers. Predictions were based on probability comparisons between current and never smokers. **c)** Boxplots showing AHRR (cg05575921) methylation levels in the FTC OLD EPICv2 samples. Stratified by reference (self-reported) smoking status (panels) and predicted smoking status (x-axis). Correctly predicted cases are shown in red; misclassified cases in blue.


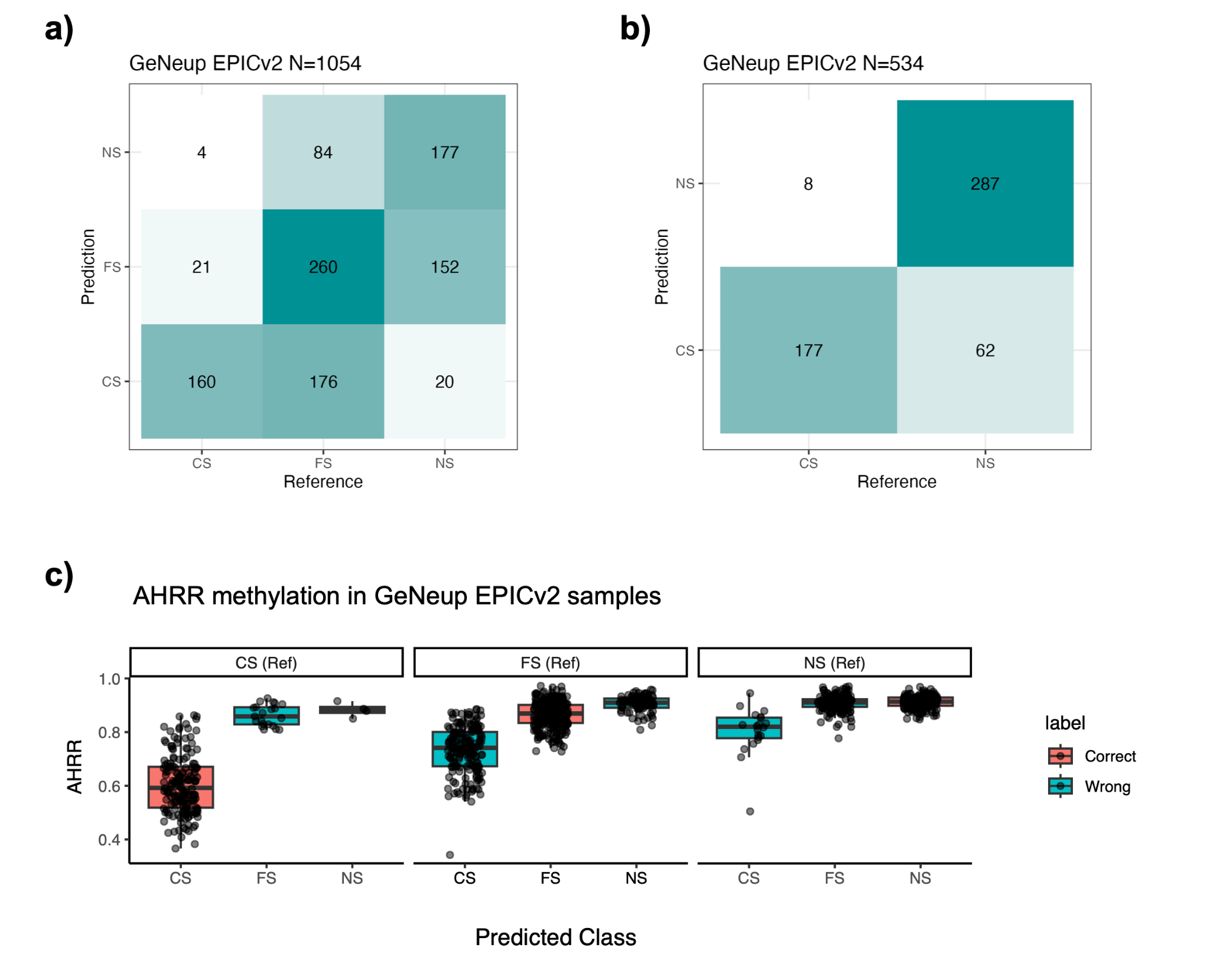


**Figure S9 Validation in GeNeup EPICv2 samples. a)** Confusion matrix for EpiSmokEr2 applied to 1054 GeNeup EPICv2 samples. Numbers represent sample counts with reference (self-reported) smoking status (x-axis) versus predicted status (y-axis). **b)** the same as a), but excluding self-reported former smokers. Predictions were based on probability comparisons between current and never smokers. **c)** Boxplots showing AHRR (cg05575921) methylation levels in the GeNeup EPICv2 samples. Stratified by reference (self-reported) smoking status (panels) and predicted smoking status (x-axis). Correctly predicted cases are shown in red; misclassified cases in blue.


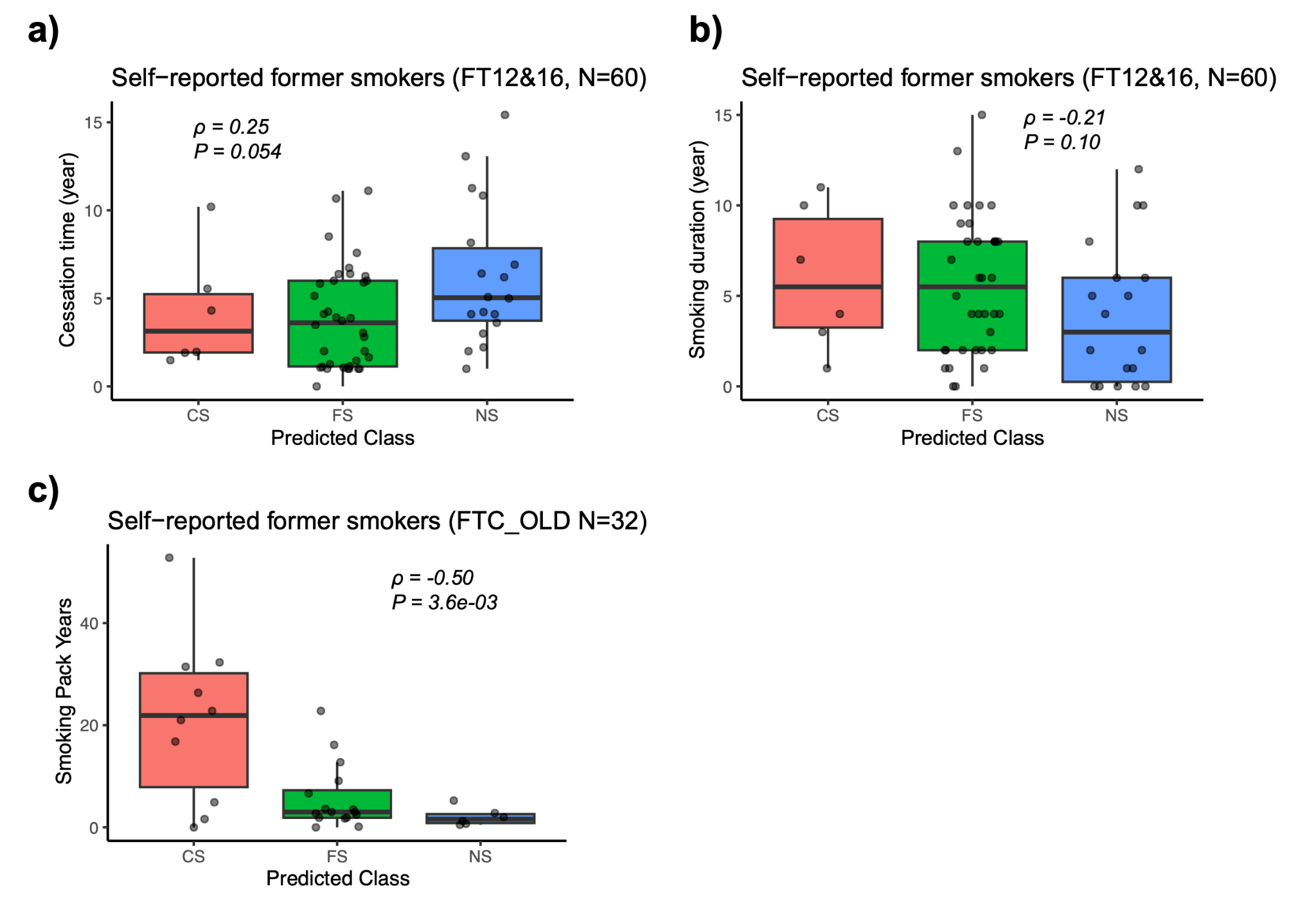


**Figure S10 Smoking history of self-reported former smokers in FTC.** **a)** Boxplot showing the time since cessation (y-axis) of self-reported former smokers in the FT12 and FT16 cohort, grouped by predicted smoking status (x-axis). **b)** Boxplot showing total smoking duration (y-axis) of self-reported former smokers in FT12 and FT16 cohort, grouped by predicted smoking status (x-axis). **c)** Boxplot showing the smoking pack-years (y-axis) of self-reported former smokers in FTC OLD cohort, grouped by predicted smoking status (x-axis). Spearman’s rank correlation coefficients (ρ) and P-values were calculated between each smoking-related measurement and the predicted smoking status, with current smokers (CS), former smokers (FS), and never smokers (NS) coded as 1, 2, and 3, respectively.


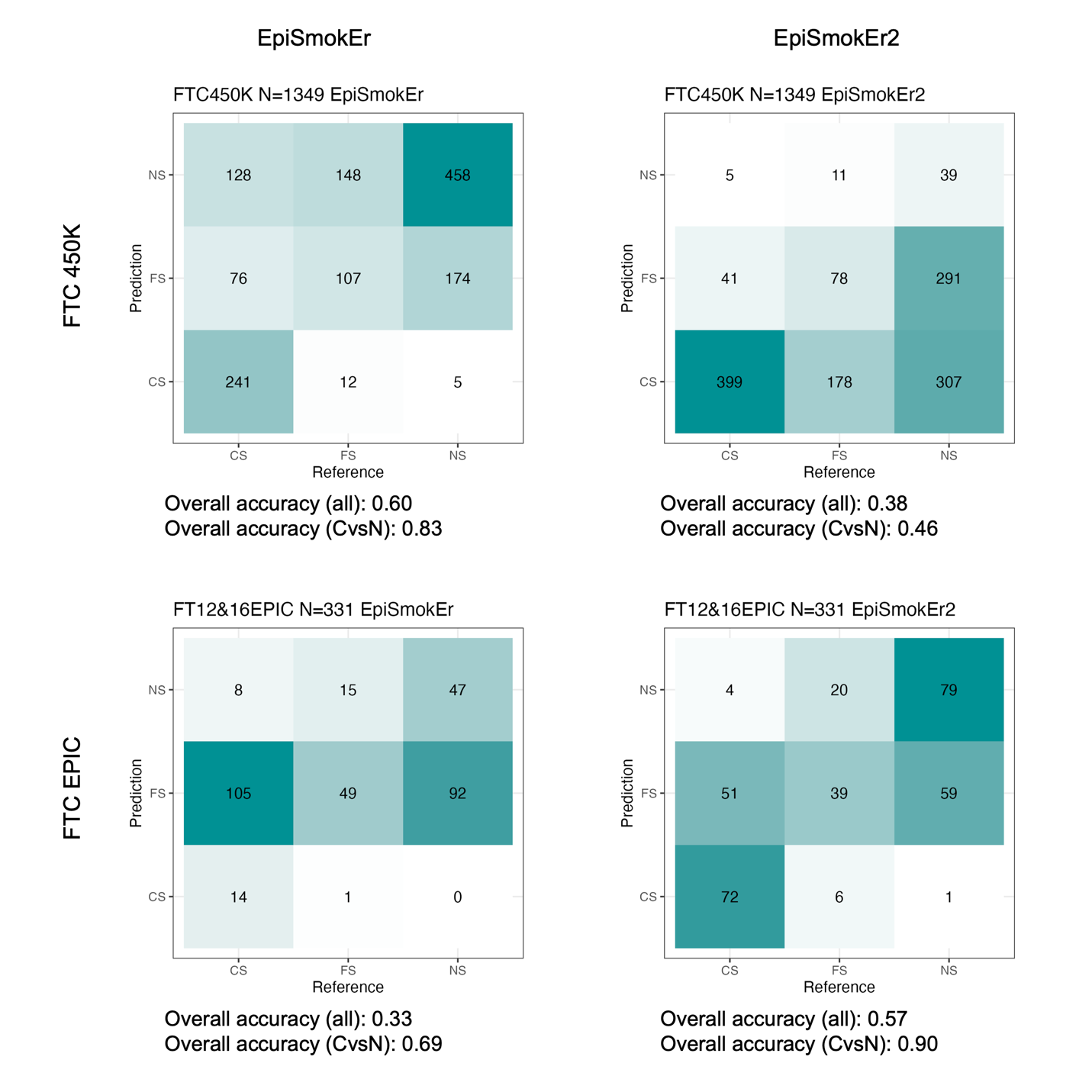


**Figure S11 Comparison of EpiSmokEr and EpiSmokEr2 performance on 450K and EPIC datasets.** Confusion matrices for EpiSmokEr and EpiSmokEr2 applied to 1,349 FTC 450K samples and 331 FT12&16 EPIC samples. Numbers represent sample counts with reference (self-reported) smoking status (x-axis) versus predicted status (y-axis). Overall classification accuracy for all three smoking categories, as well as for current and never smokers only, is reported below each matrix. Abbreviations: CS, current smoker; FS, former smoker; NS, never smoker.


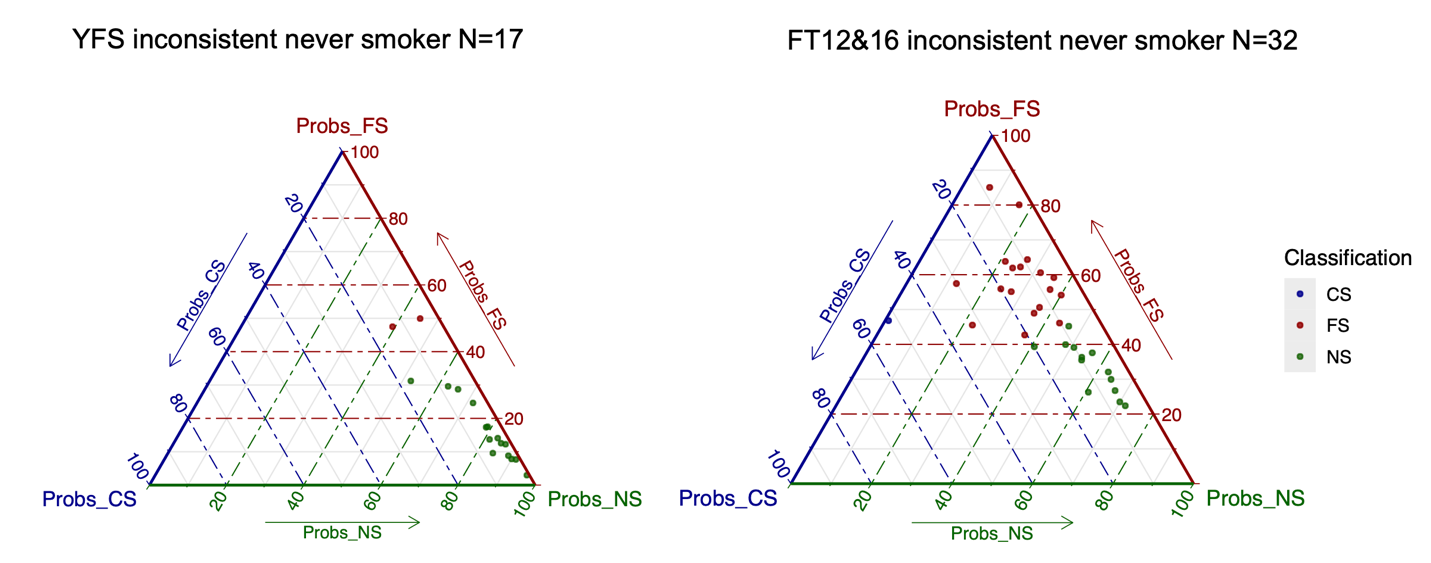


**Figure S12 EpiSmokEr2 classification of self-reported never smokers with inconsistent smoking histories.** Ternary plots showing the estimated probabilities of current (blue), former (red), and never (green) smoker status for 17 YFS and 32 FTC individuals who self-reported as never smokers but had previously reported smoking or documented passive exposure. Points were colored by the smoking category assigned by EpiSmokEr2. CS, current smoker; FS, former smoker; NS, never smoker. Abbreviations: CS, current smoker; FS, former smoker; NS, never smoker.


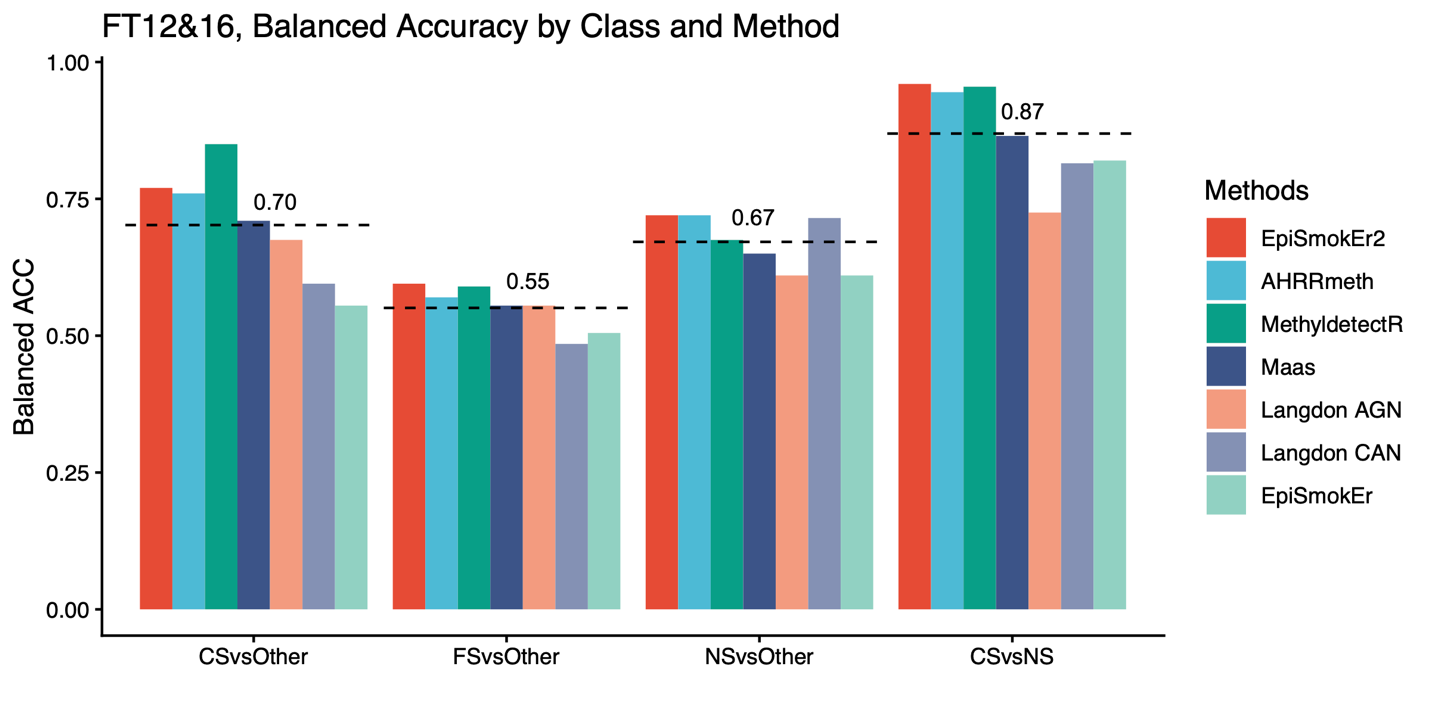


**Figure S13 Comparison of EpiSmokEr2 with DNAm-based smoking scores.** Barplot showing the balanced accuracy (defined as the mean of sensitivity and specificity) for predicting each class with seven methods: EpiSmokEr2, AHRR methylation, the MethyldetectR smoking score, Maas et al., Langdon et al. (candidate and agnostic model), and the original EpiSmokEr method. Self-reported status was used as reference. CSvsOther, FSvsOther, and NSvsOther correspond to one-vs-rest evaluations in the 3-class classification. CSvsNS corresponds to 2-class classification of current versus never smokers. The black dashed line indicates the mean balanced accuracy per smoking category. Colors indicate different methods. Abbreviations: CS, current smoker; FS, former smoker; NS, never smoker.


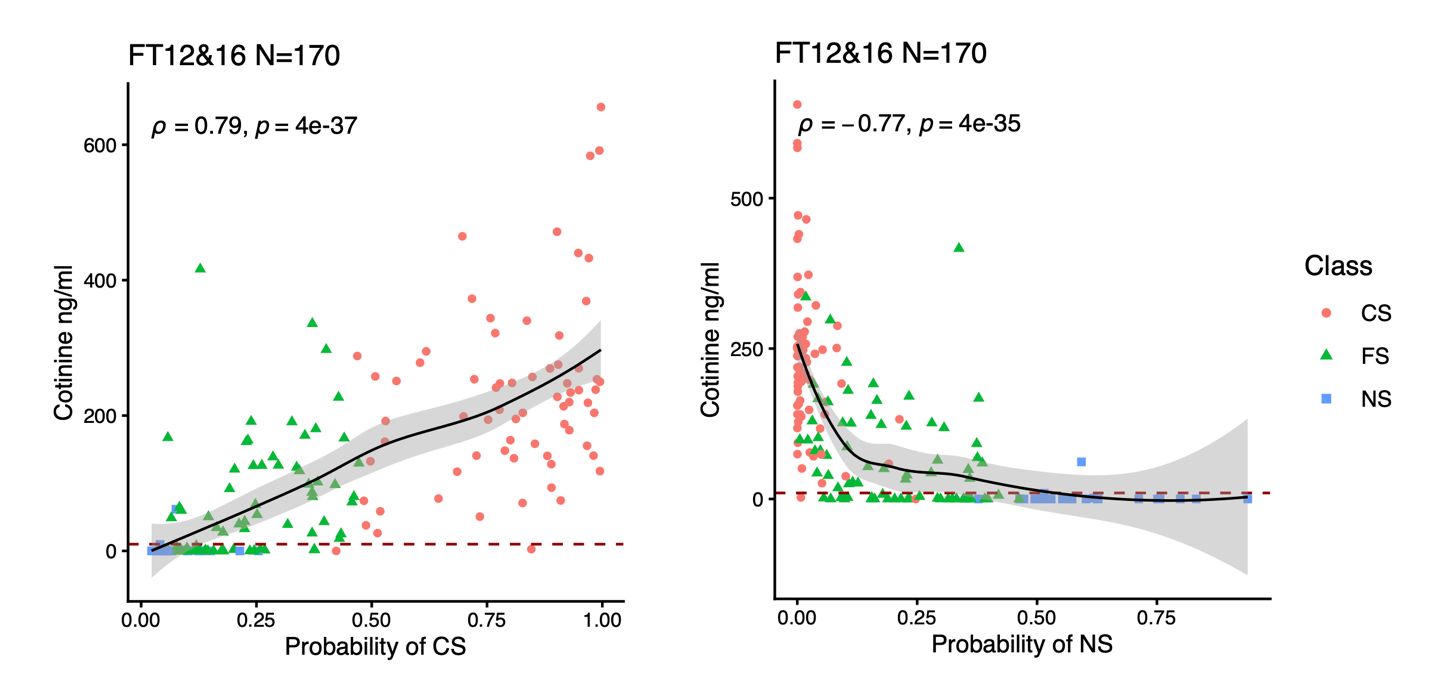


**Figure S14 Correlation between EpiSmokEr2 smoking probability and cotinine levels.** Scatterplot showing cotinine levels (y-axis) versus EpiSmokEr2-derived smoking probabilities (x-axis) in predicted current, former and never smokers. The red dashed line (10ng/ml) indicates the threshold above which individuals are considered to have smoked within 24 hours before blood draw. Spearman’s rank correlation coefficients (ρ) and P-values were calculated to assess the association between cotinine levels and EpiSmokEr2 smoking probabilities. Left: x-axis indicates the probabilities of being a current smoker; Right: x-axis indicates the probabilities of being a never smoker.

Supplementary Table

**Table S1 Performance metrics on different sex**

| Dataset | F/M | Sensitivity | | Specificity | | Overall accuracy | |
| --- | --- | --- | --- | --- | --- | --- | --- |
|  |  | Female | Male | Female | Male | Female | Male |
| FT12&16 EPIC | 164/167 |  |  |  |  | 0.6 | 0.55 |
| CS vs. others | 59/68 | 0.49 | 0.63 | 0.99 | 0.94 |  |  |
| FS vs. others | 26/39 | 0.65 | 0.56 | 0.61 | 0.56 |  |  |
| NS vs. others | 79/60 | 0.66 | 0.45 | 0.87 | 0.88 |  |  |
| CS vs. NS |  | 0.76 | 0.88 | 0.99 | 0.93 | 0.89 | 0.91 |
|  |  |  |  |  |  |  |  |
| FTC OLD EPIC | 641/60 |  |  |  |  | 0.6 | 0.3 |
| CS vs. others | 68/27 | 0.78 | 0.11 | 0.93 | 0.89 |  |  |
| FS vs. others | 78/2 | 0.32 | 0 | 0.68 | 0.45 |  |  |
| NS vs. others | 495/31 | 0.61 | 0.48 | 0.76 | 0.8 |  |  |
| CS vs. NS |  | 0.85 | 0.41 | 0.92 | 0.84 | 0.91 | 0.64 |
|  |  |  |  |  |  |  |  |
| FTC OLD EPICv2 | 145/78 |  |  |  |  |  |  |
| CS vs. others | 35/17 | 0.71 | 0.53 | 0.94 | 0.75 | 0.57 | 0.53 |
| FS vs. others | 31/38 | 0.55 | 0.61 | 0.64 | 0.6 |  |  |
| NS vs. others | 79/23 | 0.52 | 0.39 | 0.79 | 0.89 |  |  |
| CS vs. NS |  | 0.8 | 0.76 | 0.92 | 0.78 | 0.89 | 0.78 |

Note: Predicted smoking status was compared with self-reported smoking status. For each smoking status category, the sensitivity and specificity values were calculated by comparing that one with the other two categories (3-class classification), and current to never smokers (2-class classification). Overall accuracy is the proportion of all correctly classified samples out of the total number of samples. F/M column indicates the number of female/male in the corresponding category. CS=current smoker; FS=former smoker; NS=never smoker.
