# Supplementary material for: EpiSmoker2: a robust epigenetic classifier for smoking status inference using Illumina EPIC methylation data
Source: Epigenomics. Author manuscript; Available in PMC 2026 Mar 12. (PMC12962688; doi:10.1080/17501911.2026.2630841)
Supplement: Supp 2 [file NIHMS2149663-supplement-Supp_2.docx]

Supplementary methods

Description and data processing of each cohort

**The Young Finns Study (YFS)**: The Cardiovascular Risk in Young Finns Study is a Finnish prospective cohort study launched in 1980, enrolling approximately 3,600 children and adolescents aged 3–18 [1,2]. It has conducted multiple follow-ups into adulthood, integrating comprehensive assessments of lifestyle, psychosocial, biological, and socioeconomic variables to evaluate early predictors of cardiovascular disease development.

Illumina .idat files were processed with the Bioconductor package *minfi* v1.46.0 [3]. Probes with SNPs were removed using the *dropLociWithSnps* function from *minfi* with default setting. We further removed cross-reactive probes and probes on chromosome X and Y. Then, we used the Illumina definition of beta-values and derived P-values of detection for the rest of probes by comparing the total intensity U+M to that of the background distribution (given by negative control probes), as implemented in *minfi*. Beta-values with P-values of detection greater than 0.05 were set to NA. We retained samples with less than 5% missing values and probes with less than 1% missing values. 9 samples were removed, 8 of them had technical replicates. The missing beta values were then imputed with the impute.knn function (using k = 5) in R. Type-2 probe bias was corrected using Beta MIxture Quantile dilation (BMIQ) [4]. All this resulted in a 770,196 probes times 1,445 samples data matrix. Based on principal component analyses, we found a significant slide/beadchip effect. Therefore we used ComBat [5] on M-values (logit2 of beta-values) to correct for the slide effect and then converted them back to beta-values for downstream analysis.

**The Finnish Twin Cohort (FTC)**: The Finnish Twin Cohort was established in 1974. It is a population-based longitudinal study focusing on the relationship between genetics, environment, and human health and behavior. Comprising three distinct cohorts-the Older Twin Cohort [6] with a baseline survey in 1975, and the FinnTwin12 (FT12) [7] and FinnTwin16 (FT16) [8] initiated in the 1990s.

The DNA methylation data were pre-processed in the R package meffil [9]. Bad quality samples were excluded based on the following criteria: i) sex mismatch, ii) median methylation vs. unmethylated signal>3 standard deviations (SD), iii) failed control probe metrics and if>20% of probes per sample had iv) detection p value>0.01 and v) bead number<3. To remove technical variation between the samples, functional normalization including the control probe principal components was performed, followed by bad quality probe removal: i) probes with detection p value > 0.01 in more than 20% samples, ii) bead number<3 in more than 20% samples, iii) sex chromosome probes and iv) cross-reactive and ambiguously mapped probes as well as probes on polymorphic CpGs [10,11]. BMIQ normalization implemented in the R package *watermelon* [12] was then performed to adjust for type 2 probe bias. We processed EPIC and EPICv2 data with the same procedure. The data pre-processing resulted in a 765,385 probes times 1,326 samples EPIC data matrix, and a 928,635 probes times 358 samples EPICv2 data matrix.

FT12 subcohort interview as young adults: questions from the Semi-Structured Assessment for the Genetics of Alcohol (SSAGA) interviews [13]:

We determined the smoking categories based on the following

• Have you ever smoked cigarettes, cigars, pipes every day for at least a month?

• Have you smoked a total of 100 cigarettes over your lifetime?

• Have you ever tried to stop (smoking/using) cigarettes?

• What was the longest time you could be without cigarettes?

• How old were you when you used tobacco for the last time?

We determined never-smokers as those individuals who have never smoked cigarettes, pipes, or cigars, and have not smoked more than 100 cigarettes altogether. Moreover, we have classified individuals as former-smokers if they have made an attempt to quit smoking and their longest abstinence period was more than 3 months. In addition, their current age should be higher than the age at which they last smoked cigarettes. Any individuals not meeting the criteria for never-smokers or former-smokers were classified as current-smokers.

FT16ALK subcohort of young adult twins selected for pairwise discordance in alcohol use: Similarly to FT12, we determined the smoking categories based on the SSAGA interview, using the following questions:

• Have you ever smoked cigarettes, cigars, pipes every day for at least a month?

• Have you ever tried to stop (smoking/using) cigarettes?

• How old were you when you used tobacco for the last time?

Similarly, we categorized individuals as never-smokers, who have never smoked cigarettes, pipes, or cigars. Among the remaining individuals, former-smokers were identified as individuals who have ever tried to quit cigarette smoking, and their current age is higher than the age when they smoked their last cigarettes. We categorized the rest of the individuals as current-smokers.

FTC OLD cohort: Smoking status was derived from several FTC sub-studies conducted at different time points (ranging from the mid-1990s to late 2010s) [6,14]. For these sub-studies, smoking information was obtained at the time of blood sampling, either via study-specific questionnaires or structured interviews administered as part of the sub-study protocol. These data were used as the primary source for defining smoking status at the time of biosample collection. In addition, the Older Finnish Twin Cohort includes repeated baseline and follow-up questionnaires administered to cohort members since 1975 (notably in 1975, 1981, 1990, and 2011–2012), which systematically assessed smoking history and current smoking behaviour. They were used to impute missing smoking status for participants whose sub-study records did not include smoking information. In such cases, smoking-status (N=240) was inferred from the questionnaire completed closest to the sampling date within 10 years. Across data sources, smoking status was harmonized into standard categories of never smoker, former smoker, and current smoker, following established Finnish Twin Cohort conventions.

**The GuLF Study (GuLF)**: The GuLF Study is a prospective U.S. cohort designed to examine health effects of the 2010 *Deepwater Horizon* oil spill disaster [15]. The analytic samples included in this study were men, aged 21-65 years, who participated in oil spill response and cleanup efforts. The sample selection prioritized those with more biological samples (e.g., urine or serum) and more follow-up data.

In total, DNAm of 1,584 samples from 1,545 participants were measured with Illumina Infinium MethylationEPIC Beadchip using whole blood samples. A total of 1,555 samples for 834,183 CpGs passed QC: detection P value <1x10^-6^, number of beads ≥3, outlier samples or CpGs based on the distribution of total intensity plots, and Bisulfite conversion efficiency > 4000, CpG coverage per sample or per CpG> 0.95%. We estimated cell type proportions with the Houseman method [16] and surrogate variables to adjust for unknown technical artefacts implemented in the R package *Enmix* [17]. The following preprocessing steps were performed: (1) Background correction using *ENmix* method, (2) dye bias correction using RELIC method, (3) inter-array quantile normalization, and (4) probe type bias adjustment using Regression on Correlated Probes (RCP) method. For each CpG the outlier data points (beta value outside of 3IRQ range from mean) were excluded; missing values were imputed using KNN method. After removing duplicate samples and those lacking smoking information, 1,475 samples remained for downstream analysis.

We used the variable “EN_LIFESMOKE” indicating the smoking status. The variable is defined as lifetime cigarette smoking (Current/Former/Never) that was derived from two questions: (Question 1) Have you smoked at least 100 cigarettes in your entire life? Do not include cigars or marijuana. (Yes/No/Don’t know/Refused) (Question 2) Do you now smoke cigarettes? (Every day/Some days/Not at all/Don’t know/Refused). Classifications were made as follows: If participants responded, "Don’t know" or "Refused" to either questionnaire item they were classified as missing. If participants responded "No" to Question 1 they were classified as never smokers. If participants responded "Yes" to Question 1 and "Not at all" to Question 2 they were classified as former smokers. If participants responded "Yes" to Question 1 and "Every day" or "Some days" to Question 2 they were classified as current smokers.

**TwinsUK**: The TwinsUK cohort (UK Adult Twin Registry) is a longitudinal resource established in 1992 at King’s College London, initially focused on osteoporosis and rheumatologic traits [18,19]. It now comprises over 12,000–14,000 volunteer adult twin pairs (both monozygotic and dizygotic), predominantly female, aged over 18. The study features repeated questionnaire and clinical visits, extensive biobanking, and a broad array of phenotypic and multi-omics data—providing an exceptional platform for investigating genetic and environmental determinants of health, ageing, and complex disease.

Fasting whole blood DNAm was profiled using the EPICv1 array and DNAm signals were pre-processed in R. Briefly, the *ENmix* package [17] was used for quality control of the data, and the minfi package was used to exclude samples with median methylated and unmethylated signal ratio < 10.5. Background correction, dye bias correction and quantile normalization were performed with Enmix. Underperforming probes, outlier samples, signals with detP > 0.000001 and signals with nbead < 3 were excluded from the analysis. Methylation beta-values were estimated adjusting for array probe type bias with the Regression on Correlated Probes (RCP) method.

Smoking status was derived from repeated questionnaire data collected across multiple study waves. Responses were harmonized by previous investigators to obtain the most consistent classification possible. Participants were categorized as current smokers if they reported active smoking at the time of blood sampling and DNA methylation profiling, and as never smokers if they consistently reported no history of smoking. The former smoker category was defined for individuals who reported having quit smoking.

**Exploring the genetics of neuropathic pain (GeNeup)**: GeNeup is a Norwegian patient cohort consisting of 1,146 patients aged 18-70 with peripheral neuropathy. Patients were recruited between 2018 and 2023 from five Norwegian university hospitals. The study includes comprehensive questionnaire data regarding background information, pain and psychosocial information, including the Norwegian versions of Chronic Pain Grade Scale (CPG), Brief Pain Inventory (BPI), EuroQol EQ-5D, Bergen Insomnia Scale, Pain Catastrophizing Scale, painDETECT and the Self-completed Leeds Assessment of Neuropathic Symptoms and Signs (S-LANSS), in addition to biological samples and clinical characterization.

DNAm data was preprocessed using RnBeads. Probes with SNPs (n = 9,981) and poor-performing probes with a detection p-value >0.01 (n = 34,725) were removed. Background correction was performed using the ENmix exponential-truncated-normal out-of-band (oob) method[17], followed by beta-mixture quantile (BMIQ) normalization[4]. Non-CpG probes (n = 2,324), cross-reactive probes (n = 27,059, probes on the sex chromosomes (n = 21,886) were then removed.

Current, former and never smoker definition:

Question about smoking habits.

- NS = “I have never smoked”.

- FS = “I used to smoke sometimes or daily”

- CS = “I currently smoke sometimes or daily”

Of the 520 former smokers we have information about the age the former smokers stopped smoking for n = 129, for n = 408 this information is missing. Of the information we do have, all FSs except 10 stopped smoking more than 1 year ago.

**Comparison to score-based methods**

We compared our EpiSmokEr2 with five established DNAm score-based smoking predictors: AHRR methylation, the MethyldetectR smoking score [20], Maas et al. [21], Langdon et al. (candidate and agnostic model) [22], and the original EpiSmokEr method [23]. All scores were calculated according to the procedures described in the respective publications. Missing CpGs in our data were imputed using the mean methylation level across YFS individuals.

To enable categorical comparison with EpiSmokEr2, optimal thresholds were derived for each score-based method using the YFS data. Using the *pROC* R package [24], receiver operating characteristic (ROC) curves were generated with self-report smoking status as the response variable, and each DNAm score as the predictor. Thresholds were selected by minimising the Euclidean distance between the ROC curve and the (0, 1) point, corresponding to the optimal trade-off between sensitivity and specificity.

Performance was evaluated using balanced accuracy (mean of sensitivity and specificity) for both 3-class (current vs. others, former vs. others, never vs. others) and 2-class (current vs. never) classification.

References

[1] Åkerblom HK, Uhari M, Pesonen E, et al. Cardiovascular Risk in Young Finns. Annals of Medicine. 1991;23(1):35–39. doi: 10.3109/07853899109147928

[2] Raitakari OT, Juonala M, Ronnemaa T, et al. Cohort Profile: The Cardiovascular Risk in Young Finns Study. International Journal of Epidemiology. 2008;37(6):1220–1226. doi: 10.1093/ije/dym225

[3] Aryee MJ, Jaffe AE, Corrada-Bravo H, et al. Minfi: a flexible and comprehensive Bioconductor package for the analysis of Infinium DNA methylation microarrays. Bioinformatics. 2014;30(10):1363–1369. doi: 10.1093/bioinformatics/btu049

[4] Teschendorff AE, Marabita F, Lechner M, et al. A beta-mixture quantile normalization method for correcting probe design bias in Illumina Infinium 450 k DNA methylation data. Bioinformatics. 2013;29(2):189–196. doi: 10.1093/bioinformatics/bts680

[5] Johnson WE, Li C, Rabinovic A. Adjusting batch effects in microarray expression data using empirical Bayes methods. Biostatistics. 2007;8(1):118–127. doi: 10.1093/biostatistics/kxj037

[6] Kaprio J, Bollepalli S, Buchwald J, et al. The Older Finnish Twin Cohort — 45 Years of Follow-up. Twin Res Hum Genet. 2019;22(4):240–254. doi: 10.1017/thg.2019.54

[7] Rose RJ, Salvatore JE, Aaltonen S, et al. FinnTwin12 Cohort: An Updated Review. Twin Res Hum Genet. 2019;22(5):302–311. doi: 10.1017/thg.2019.83

[8] Kaidesoja M, Aaltonen S, Bogl LH, et al. FinnTwin16: A Longitudinal Study from Age 16 of a Population-Based Finnish Twin Cohort. Twin Res Hum Genet. 2019;22(6):530–539. doi: 10.1017/thg.2019.106

[9] Min JL, Hemani G, Davey Smith G, et al. Meffil: efficient normalization and analysis of very large DNA methylation datasets. Hancock J, editor. Bioinformatics. 2018;34(23):3983–3989. doi: 10.1093/bioinformatics/bty476

[10] Chen Y, Lemire M, Choufani S, et al. Discovery of cross-reactive probes and polymorphic CpGs in the Illumina Infinium HumanMethylation450 microarray. Epigenetics. 2013;8(2):203–209. doi: 10.4161/epi.23470

[11] Zhou W, Laird PW, Shen H. Comprehensive characterization, annotation and innovative use of Infinium DNA methylation BeadChip probes. Nucleic Acids Res. 2016;gkw967. doi: 10.1093/nar/gkw967

[12] Pidsley R, Y Wong CC, Volta M, et al. A data-driven approach to preprocessing Illumina 450K methylation array data. BMC Genomics. 2013;14(1):293. doi: 10.1186/1471-2164-14-293

[13] Bucholz KK, Cadoret R, Cloninger CR, et al. Semi-Structured Assessment for the Genetics of Alcoholism [Internet]. 1994 [cited 2025 July 1]. doi: 10.1037/t03926-000 Available from: https://doi.apa.org/doi/10.1037/t03926-000

[14] Kaprio J. The Finnish Twin Cohort Study: an update. Twin Res Hum Genet. 2013;16(1):157–162. doi: 10.1017/thg.2012.142

[15] Kwok RK, Engel LS, Miller AK, et al. The GuLF STUDY: A Prospective Study of Persons Involved in the *Deepwater Horizon* Oil Spill Response and Clean-Up. Environ Health Perspect. 2017;125(4):570–578. doi: 10.1289/EHP715

[16] Houseman EA, Accomando WP, Koestler DC, et al. DNA methylation arrays as surrogate measures of cell mixture distribution. BMC Bioinformatics. 2012;13(1):86. doi: 10.1186/1471-2105-13-86

[17] Xu Z, Niu L, Li L, et al. ENmix: a novel background correction method for Illumina HumanMethylation450 BeadChip. Nucleic Acids Res. 2016;44(3):e20–e20. doi: 10.1093/nar/gkv907

[18] Moayyeri A, Hammond CJ, Valdes AM, et al. Cohort Profile: TwinsUK and Healthy Ageing Twin Study. International Journal of Epidemiology. 2013;42(1):76–85. doi: 10.1093/ije/dyr207

[19] Verdi S, Abbasian G, Bowyer RCE, et al. TwinsUK: The UK Adult Twin Registry Update. Twin Res Hum Genet. 2019;22(6):523–529. doi: 10.1017/thg.2019.65

[20] Hillary RF, Marioni RE. MethylDetectR: a software for methylation-based health profiling. Wellcome Open Res. 2021;5:283. doi: 10.12688/wellcomeopenres.16458.2

[21] BIOS Consortium, Maas SCE, Vidaki A, et al. Validated inference of smoking habits from blood with a finite DNA methylation marker set. Eur J Epidemiol. 2019;34(11):1055–1074. doi: 10.1007/s10654-019-00555-w

[22] Langdon RJ, Yousefi P, Relton CL, et al. Epigenetic modelling of former, current and never smokers. Clin Epigenet. 2021;13(1):206. doi: 10.1186/s13148-021-01191-6

[23] Bollepalli S, Korhonen T, Kaprio J, et al. EpiSmokEr: a robust classifier to determine smoking status from DNA methylation data. Epigenomics. 2019;11(13):1469–1486. doi: 10.2217/epi-2019-0206

[24] Robin X, Turck N, Hainard A, et al. pROC: an open-source package for R and S+ to analyze and compare ROC curves. BMC Bioinformatics. 2011;12(1):77. doi: 10.1186/1471-2105-12-77
